# Molecular Targets of Manganese-Induced Neurotoxicity: A Five-Year Update

**DOI:** 10.3390/ijms22094646

**Published:** 2021-04-28

**Authors:** Alexey A. Tinkov, Monica M. B. Paoliello, Aksana N. Mazilina, Anatoly V. Skalny, Airton C. Martins, Olga N. Voskresenskaya, Jan Aaseth, Abel Santamaria, Svetlana V. Notova, Aristides Tsatsakis, Eunsook Lee, Aaron B. Bowman, Michael Aschner

**Affiliations:** 1 Laboratory of Ecobiomonitoring and Quality Control, Yaroslavl State University, 150003 Yaroslavl, Russia; tinkov.a.a@gmail.com; 2 Laboratory of Molecular Dietetics, Department of Neurological Diseases and Neurosurgery, Department of Analytical and Forensic Toxicology, IM Sechenov First Moscow State Medical University (Sechenov University), 119435 Moscow, Russia; vos-olga@yandex.ru (O.N.V.); jaol-aas@online.no (J.A.); tsatsaka@uoc.gr (A.T.); 3 Department of Molecular Pharmacology, Albert Einstein College of Medicine, Bronx, NY 10461, USA; monica.paoliello@einsteinmed.org (M.M.B.P.); airton.dacunhamartinsjunior@einsteinmed.org (A.C.M.); 4 Graduate Program in Public Health, Center of Health Sciences, State University of Londrina, Londrina, PR 86038-350, Brazil; 5 Department of Medical Elementology, Peoples’ Friendship University of Russia (RUDN University), 117198 Moscow, Russia; gman65@mail.ru; 6 World-Class Research Center “Digital Biodesign and Personalized Healthcare”, IM Sechenov First Moscow State Medical University (Sechenov University), 119435 Moscow, Russia; skalny3@microelements.ru; 7 Laboratory of Medical Elementology, KG Razumovsky Moscow State University of Technologies and Management, 109004 Moscow, Russia; 8 Research Department, Innlandet Hospital Trust, P.O. Box 104, 2381 Brumunddal, Norway; 9 Laboratorio de Aminoácidos Excitadores, Instituto Nacional de Neurología y Neurocirugía, SSA, Mexico City 14269, Mexico; absada@yahoo.com; 10 Institute of Bioelementology, Orenburg State University, 460018 Orenburg, Russia; snotova@mail.ru; 11 Federal Research Centre of Biological Systems and Agro-technologies of the Russian Academy of Sciences, 460000 Orenburg, Russia; 12 Laboratory of Toxicology, Medical School, University of Crete, Voutes, 700 13 Heraklion, Greece; 13 Department of Pharmaceutical Sciences, Florida A&M University, Tallahassee, FL 32307, USA; eunsook.lee@famu.edu; 14 School of Health Sciences, Purdue University, West Lafayette, IN 47906, USA; bowma117@purdue.edu

**Keywords:** manganese, neurotoxicity, neuroinflammation, apoptosis, cell signaling

## Abstract

Understanding of the immediate mechanisms of Mn-induced neurotoxicity is rapidly evolving. We seek to provide a summary of recent findings in the field, with an emphasis to clarify existing gaps and future research directions. We provide, here, a brief review of pertinent discoveries related to Mn-induced neurotoxicity research from the last five years. Significant progress was achieved in understanding the role of Mn transporters, such as SLC39A14, SLC39A8, and SLC30A10, in the regulation of systemic and brain manganese handling. Genetic analysis identified multiple metabolic pathways that could be considered as Mn neurotoxicity targets, including oxidative stress, endoplasmic reticulum stress, apoptosis, neuroinflammation, cell signaling pathways, and interference with neurotransmitter metabolism, to name a few. Recent findings have also demonstrated the impact of Mn exposure on transcriptional regulation of these pathways. There is a significant role of autophagy as a protective mechanism against cytotoxic Mn neurotoxicity, yet also a role for Mn to induce autophagic flux itself and autophagic dysfunction under conditions of decreased Mn bioavailability. This ambivalent role may be at the crossroad of mitochondrial dysfunction, endoplasmic reticulum stress, and apoptosis. Yet very recent evidence suggests Mn can have toxic impacts below the no observed adverse effect of Mn-induced mitochondrial dysfunction. The impact of Mn exposure on supramolecular complexes SNARE and NLRP3 inflammasome greatly contributes to Mn-induced synaptic dysfunction and neuroinflammation, respectively. The aforementioned effects might be at least partially mediated by the impact of Mn on α-synuclein accumulation. In addition to Mn-induced synaptic dysfunction, impaired neurotransmission is shown to be mediated by the effects of Mn on neurotransmitter systems and their complex interplay. Although multiple novel mechanisms have been highlighted, additional studies are required to identify the critical targets of Mn-induced neurotoxicity.

## 1. Introduction

Manganese (Mn) is an essential metal that is involved in a variety of physiological processes [1]. Mn naturally occurs in the Earth’s crust, predominantly as the ^55^Mn isotope, although a total of 18 isotopes have been described [2]. In biological systems, Mn exists in two oxidation states Mn^2+^ and Mn^3+^ that mediate redox cycling of Mn, which is involved in biological effects of the metal, including the Fenton reaction, transferrin-mediated transport, interference, as well as interference with other divalent metals (Mg^2+^, Fe^2+^), to name a few [3]. At the same time, in the environment Mn may exist in other positive (^4+^, ^5+^, and ^6+^) and even negative (^3-^) oxidation states. Due to its chemistry Mn exists in multiple inorganic and organic species. The most common inorganic species include oxides (dioxide, MnO_2_, and tetraoxide, Mn_3_O_4_), chloride (MnCl_2_), sulfate (MnSO_4_), manganese phosphate (MnPO_4_), carbonate (MnCO_3_), silicate (MnSiO_3_), etc. [4]. Among organic Mn species, methylcyclopentadienyl Mn tricarbonyl (MMT) as a gasoline additive, and Maneb and Mancozeb as pesticides/fungicides may be considered as significant health hazard due to high risk of overexposure [5]. In addition to anthropogenic sources of Mn into the environment, natural sources including soil erosion may also contribute to Mn emissions [4].

In addition to regulation of redox homeostasis, energy metabolism, and regulation of urea cycle [1], Mn is also known to play a significant role in regulation of neuronal development [6]. Alteration of these processes, under Mn deficiency or excess conditions, may result in severe metabolic dysfunction. However, the case for dietary Mn deficiency appears to be extremely rare in humans due to high levels of Mn in dietary products [7,8]. In contrast, Mn overexposure, which is far more common, may cause brain-associated disorders [9]. Studies on neurological outcomes have been carried out in populations living in areas near industrial activities, including former and active Mn alloys plants, and Mn ore processing plants [10,11,12,13], among others.

Mn exposure was shown to be associated with a number of adverse neurological effects [14]. Multiple. Multiple studies have demonstrated significant association between Mn exposure and neurodegenerative diseases [15]. Specifically, a recent meta-analysis demonstrated a significantly higher circulating Mn levels in patients with Parkinson’s disease [16]. Elevation of Mn levels was also observed in amyotrophic lateral sclerosis [17], whereas the association between Mn overexposure and Alzheimer’s disease appears to be inverse [18]. However, the time of assessment may have a significant impact on the outcome of such studies due to Mn excretion from the organism.

Mn neurotoxicity is also known to affect neurodevelopment [19]. Specifically, maternal or early-life Mn exposure was shown to be associated with poorer cognitive and behavioral performance in children under six years old [20]. However, in view of the essentiality of physiological Mn levels and its toxicity upon overexposure [21], the association between Mn and adverse neurodevelopmental outcome may be U-shaped [22]. The potential contribution of Mn exposure to attention deficit hyperactivity disorder (ADHD) was also demonstrated [23].

In view of the significant neurological effects of Mn exposure, the mechanisms of Mn-induced neurotoxicity have been extensively studied. Key mechanisms include neuroinflammation, impaired calcium homeostasis [24], dysregulation of mitochondrial function and redox homeostasis [25], altered proteostasis [26], impaired microRNAs (miRNA) function [27], and altered neurotransmitter metabolism [28], to name a few. Additionally, reports suggest that Mn homeostasis is affected by low dose cadmium feeding [29]. However, the understanding of the intimate mechanisms of Mn neurotoxicity is rapidly evolving in view of the new data obtained.

Despite the presence of numerous outstanding reviews on Mn neurotoxicity, the summary of recent findings in the field is of particular interest in order to clarify the existing gaps and further research directions. Previous updates were provided in 2015 [30], 2016 [31], and 2018 [32]. In view of the significant progress in the field, in this paper we provide a brief review of pertinent discoveries in Mn-induced neurotoxicity research during the last five years.

## 2. Manganese Transporters

Regulation of brain Mn homeostasis is a critical mechanism for supporting the balance between Mn essentiality and toxicity [33]. Although not representing molecular targets of Mn neurotoxicity, recent data on Mn transporters are briefly reviewed herein due to their importance in regulating brain Mn levels.

### 2.1. SLC39A14 (ZIP14)

It has been proposed that modulation of ZIP14 may be involved in prevention of Mn-induced neurotoxicity [34]. A study in intestine-specific ZIP14-KO demonstrated that intestinal ZIP14 deficiency is responsible for systemic and brain Mn accumulation upon overexposure and cannot be compensated by hepatobiliary metal excretion [35]. ZIP14 deficiency in ZIP14 knock-out (KO) mice resulted in reduced Mn excretion after subcutaneous Mn administration as well as increased cerebral Mn accumulation with subsequent motor dysfunction [34]. The primary localization of Mn deposits in brain was observed in the pons and basal ganglia, including globus pallidus [36]. Mn^2+^ exposure was shown to down-regulate ZIP14 expression in HepaRG cells and subsequent Mn transport, indicative of the involvement of ZIP14 in a cytoprotective response upon Mn overexposure [37].

Clinical findings also support the role of ZIP14 in regulation of brain Mn homeostasis. A novel missense variant (c.311G  >  T; p.Ser104Ile) in the *SLC39A14* gene was found to be associated with acute dystonia and motor regression. Clinical symptoms were also associated with pallidal Mn accumulation and a predominant accumulation of the metal in cerebrospinal fluid (CSF) as compared to peripheral blood [38]. A missense variant c.1136.T in exon 7 of *SLC39A14* gene was clinically characterized by hypermanganesemia, dystonia, and iron deficiency anemia [39].

### 2.2. SLC30A10 (ZNT10)

The role of SLC30A10 as Mn transporter was verified in *SLC30A10*-knocked down worms, *Caenorhabditis elegans.* which were characterized by increased survival in response to Mn exposure [40]. Our recent studies using tissue-specific *SLC30A10* knockout mice demonstrated enterocytic *SLC30A10* expression, being indicative of the role of both liver and gastrointestinal tract in regulation of brain Mn levels in physiological conditions, whereas at higher Mn exposure brain SLC30A10 is responsible for neuroprotection [41]. Correspondingly, mice with hepatic and intestinal SLC30A10 deficiency were characterized by less severe Mn overload as compared to whole-body deficient animals thus underlining involvement of other tissues in regulation of Mn accumulation [42]. Cases of familiar mutations in *SLC30A10* or *SLC39A14* genes are characterized by systemic and cerebral Mn overload and severe neurotoxicity [43]. Specifically, a homozygous missense mutation in *SLC30A10* was characterized by increased whole blood and basal ganglia Mn levels, dystonia, polycythemia, and cirrhosis [44].

### 2.3. SLC39A8 (ZIP8)

ZIP8 along with ZIP14 have been considered as the most significant regulators of Mn uptake in brain microvascular capillary endothelial cells as compared to DMT-1 and endocytic uptake, the latter being responsible for both apical and basal transmembrane Mn^2+^ transport in blood-brain barrier cells [45]. Comparative analysis of total and liver-specific ZIP8-knockout mice demonstrated that hepatic ZIP8 plays a key role in regulation of systemic Mn homeostasis with subsequent modulation of Mn-dependent arginase and β-1,4-galactosyltransferase, as well as protein N-glycosylation [46]. Correspondingly, a mutant *SLC39A8* variant was shown to be associated with systemic cerebral atrophy, developmental delay, dystonia, Mn deficiency, as well as impaired hepatic electron transport chain complexes that may be mediated by reduced activity of the Mn-dependent β-galactosyltransferase and MnSOD [47]. Although SLC39A8 is considered as multiple metal transporter, a common *SLC39A8* missense (A391T) mutation was characterized by significantly reduced levels of serum Mn, but not other metals (Co, Cu, Zn) [48]. Along with Mn-dependent disorders, SLC39A8 deficiency is associated with birth defects, lipid disorders, cardiovascular diseases, neurological and neurodegenerative diseases, as well as inflammatory disorders [49].

## 3. Mn-Induced Alterations in Subcellular and Multicellular Biology

### 3.1. Gene Expression

Several studies have focused on genetic networks affected by Mn. specifically, evaluation of shared microarray data from Mn-treated neurons and intact cells revealed 140 upregulated and 267 down-regulated genes. Gene ontology function analysis demonstrated that the differentially expressed genes were involved mainly in chemotaxis, intercellular signaling, regulation of metabolism, and response to wounding. In turn, KEGG pathway analysis characterized cytokine-cytokine receptor interaction, apoptosis, oxidative phosphorylation, Toll-like receptor signaling pathway, and insulin signaling pathway genes as the most affected. Of these gene networks, *INSR*, *VEGFA*, *PRKACB*, *DLG4*, and *BCL2* could be considered as candidate genes associated with Mn-induced Alzheimer’s disease [50]. In another study, genome-wide sequencing of striatal samples from Mn-exposed rats (25 mg/kg MnCl_2_·4H_2_O i.p. every 48 h for a month) revealed seven down-regulated and 10 up-regulated genes. Specifically, genes involved in redox metabolism, dopamine synthesis, apoptosis, and neuronal survival including *Sgk1*, *HCRTr1*, *HspB1*, *Rem2*, *Oprd1*, *ATF5*, and *TRHr* may determine susceptibility to Mn toxicity [51]. Our earlier study demonstrated that Mn exposure (50 mg/kg MnCl_2_·4H_2_O s.c. twice a week for 20 weeks) may differentially affect genetic networks in the wild-type mice and a genetic model of Huntington’s disease (YAC128). Specifically, Mn exposure in wild-type mice affected metabolic pathways tightly linked to brain-derived neurotrophic factor (BDNF), whereas the targets of Mn exposure in YAC128 were focused upon the *Htt* gene involved in cell growth and proliferation [52]. A transcriptomic approach was used to unravel differentially expressed genes in Mn-exposed (100 μM Mn (sublethal) for 30 days) and control SH-SY5Y cells. Microarray and subsequent cluster analysis demonstrated that 1057 differentially-expressed transcripts, being predominantly involved in regulation of neuronal differentiation and development, apoptosis, and synaptic transmission [53]. Correspondingly, using zebrafish slc39a14^U801-/-^ mutants exposed to Mn (50 µM for 72 h) for analysis of differentially-expressed genes demonstrated that the genes associated with Mn neurotoxicity are associated with mitochondrial dysfunction, oxidative stress, apoptosis, lysosomal dysfunction, altered proteostasis and unfolded protein response, Ca^2+^ dyshomeostasis, as well as impaired visual phototransduction [54]. In parallel with the identified gene networks differentially expressed following acute Mn exposure (50 mM for 30 min), metal overload was also found to be associated with modulation of endoplasmic reticulum related genes and lipocalin-related (lpr) family members, thus indicating additional targets for Mn toxicity [55].

Exposure of dopaminergic neurons to Mn (150 µM for 48 h) and the Parkinson’s disease model toxin, 1-methyl-4-phenylpyridinium ion (MPP^+^), led to 694 and 603 upregulated, and 428 and 255 down-regulated genes, respectively. The differentially expressed genes were related to mitochondrial dysfunction, neuroinflammation, apoptosis, altered synaptic plasticity, impaired neurotransmission, and cytoskeleton abnormalities. However, the impact of Mn and MPP^+^ on pathways of neurogenesis and neurite outgrowth was quite different, being indicative of differences in pathogenesis of Mn- and MPP^+^-associated Parkinson’s disease [56].

Correspondingly, complex analysis of pathways affected by Mn exposure using metallomics, proteomics, gene expression, and bioinformatics demonstrated that irrespective of speciation, Mn exposure alters proteostasis, cell metabolism and signaling, immunity and inflammation, cell cycle, and neurodegeneration-associated pathways. In turn, altered neurotransmission pathways were found to be characteristic only for inorganic MnCl_2_ (1.5 mg Mn/kg i.v. for four days) but not maneb ([[2-[(dithiocarboxy) amino] ethyl]-carbamodithioato]](2-)-kS,kS′]manganese. It is proposed that the variance in effects between the studied Mn species may occur due to differences in post-translational modification of target proteins, being more pronounced in the case of maneb [57]. Metabolomics analysis revealed the impact of Mn on amino acid, glutathione, glucose, fatty acid, and purine/pyrimidine metabolism both in vivo [58] and in vitro [59], thus corresponding to the genetic analysis of the affected pathways.

Metabolomics also revealed biphasic effects of Mn exposure on metabolic pathways. Specifically, exposure to low-dose Mn (10 μM for 5 h) resulted in a significant modulation of neurotransmitter, energy, fatty acid, and amino acid metabolism with an increase in neuroprotective amino acid metabolites including creatine, phosphocreatine, phosphoserine, whereas exposure to the toxic dose (100 μM for 5 h) disrupted energy and fatty acid metabolism along with induction of cell death [60]. Correspondingly, transcriptomic analysis from the same research group demonstrated a dose-dependent change in differentially-expressed genes in response to physiological and toxic Mn exposures. Low-dose Mn exposure (10 μM Mn for 5 h) resulted in a significant increase in Golgi-residing proteins (BET1, ADAM10, ARFGAP3) gene expression, whereas high dose Mn (100 μM Mn for 5 h) exposure was shown to alter oxidative phosphorylation pathway and energy metabolism genes including *ATP6V1H*, *NDUFAF5*, and *FABP5* prior to induction of cell death [61].

### 3.2. Epigenetics

In parallel with direct effects of Mn exposure on genetic apparatus, the most recent studies have also characterized the epigenetic effects of this metal. Specifically, analysis of gene methylation in Mn-exposed SH-SY5Y cells (100 µM for 30 days) revealed differential methylation of 10,213 genes. Clustering using Database for Annotation, Visualization and Integrated Discovery (DAVID) demonstrated that hypermethylated genes are involved in metal ion binding, regulation of cytoskeleton, chromatin modification, regulation of transcription, apoptosis, and iron binding, whereas hypomethylated genes may be responsible for signal transduction, transcription, neuron differentiation and development, synaptic transmission, and MAPK signaling. It is noteworthy that certain differentially methylated genes are implicated in Parkinson’s disease pathogenesis [62]. Genome-wide analysis demonstrated that Mn exposure in mice (5 mg/kg i.p. twice a week for six weeks) resulted in altered DNA methylation in the promoter region of 226 genes involved in mitochondrial functioning, cell cycle, DNA damage repair, and ion transport, DMOG [N-(2-methoxy-2-oxoacetyl) glycine was capable of restoring methylation of certain genes [63].

In utero Mn exposure was also shown to alter placental DNA methylation of 731 CpG loci with five most affected involved in neurodevelopment, fetal development, and carcinogenesis [64]. Finally, in welders exposed to metal-containing fumes, Mn overload was associated with iNOS gene methylation and parkinsonism [65].

Mn was also shown to affect epigenetic regulation of histone acetylation. Specifically, exposure to 300 μM MnCl_2_ for 3, 6, 12, or 24 h was shown to suppress histone H3 and H4 acetylation in PC12 cells and SHSY5Y cells through up-regulation of histone deacetylases (HDAC) and inhibition of histone acetyltransferase (HAT) expression [66].

It is notable that epigenetic effects of Mn exposure may be mediated by its influence on α-synuclein overexpression and aggregation [67].

### 3.3. Cell Signaling

Mn is an essential co-factor for many kinases and phosphatases that play critical roles in cell signaling pathways. A role for alteration of cell signaling activity under conditions of Mn neurotoxicity have been reported for p53, insulin and insulin growth factor signaling, as well as AKT and mTOR signaling. Specifically, our previous study in *C. elegans* demonstrated that loss-of-function mutations in AKT1/2 and serum and glucocorticoid-regulated kinase (SGK-1) are associated with increased resistance to Mn exposure at doses of 2.5–100 mM for 1 h, being indicative of the role of these pathways in Mn toxicity [68]. At the same time, it is proposed that PI3K may mediate the effects of 200 μM Mn on AKT and mTOR and downstream signaling, also acting as Mn “sensor” [69]. Mn (1–10 μM for 24 h) was also shown to modulate insulin-IGF signaling network through increasing IGF1 and insulin levels that may mediate modulatory effects of Mn exposure on AKT. In particular, Mn^2+^ exposure was shown to potentiate p-IGFR/IR-dependent AKT phosphorylation both under physiological and supraphysiological levels, being responsible for more than 70% of Mn-induced Akt signaling in cellular models of Huntington’s disease [70]. Given the role of these pathways in cell signaling, Mn-induced modulation of PI3K/AKT/mTOR may underlie the effects of Mn (50–200 μM) on downstream signaling targets including p53. In addition, Mn was shown to activate ataxia telangiectasia mutated (ATM) kinase being responsible for p53 phosphorylation [71].

### 3.4. Neurogenesis

Environmentally relevant Mn exposure (500–800 μM for 24 h) is known to induce cytoskeletal reorganization in neurons with inhibition of neuronal differentiation and neurite outgrowth [72].

In rat hippocampal dentate gyrus exposure to 6 mg Mn/kg (5 days/week) for four weeks resulted in a significant decrease of proliferating cells, reduced cellular survival, impaired differentiation, and neurite outgrowth. In addition, impaired migration of the neuroblasts from the subgranular zone to the granule cell was also revealed [73]. Although Mn exposure was shown to reduce cell survival and neurogenesis in the olfactory bulb, although in brain subventricular zone it induced an initial increase in cell proliferation [74]. Correspondingly, in subventricular zone and rostral migratory stream exposure to 6 mg Mn/kg as MnCl_2_ once daily for four weeks significantly increased neurogenesis as evidenced by elevated number of bromodeoxyuridine-positive cells, increased GFAP-positive astrocytic stem cells, and doublecortin-positive neuroblasts. However, the observed cellular Mn overaccumulation due to Mn-induced increase in DMT1 mRNA expression was also associated with a reduction in Cu levels, thus disrupting normal neurogenesis [75].

It has also expression of antioxidant enzymes and been demonstrated that dietary (800 ppm in diet for 56 days) Mn-induced decrease of granule cell BDNF signaling through alteration of c-Fos-mediated neuronal plasticity may result in γ-aminobutyric acid (GABA)-ergic interneuron loss altogether leading to disrupted neurogenesis [76].

### 3.5. Neuroinflammation

**Figure 1:**
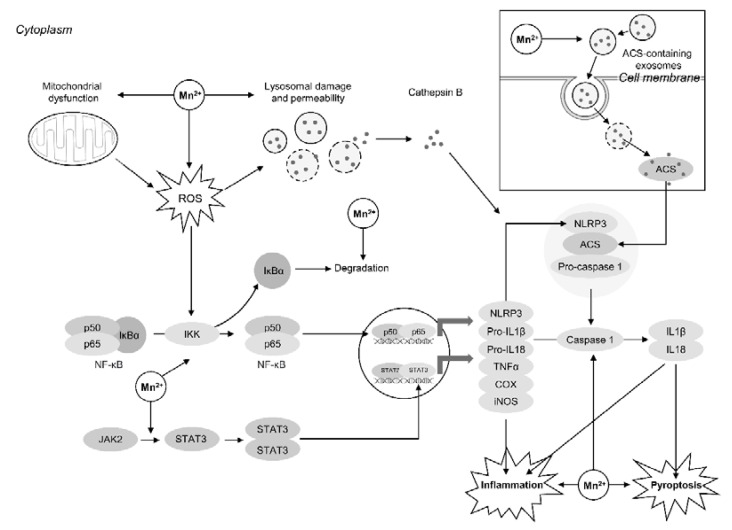
Neuroinflammatory effects of Mn exposure. NF-κB activation through a number of mechanisms plays a key role in proinflammatory effects of Mn [81]. Specifically, Mn overload significantly increased IkBα degradation ultimately resulting in NF-κB activation [82]. In addition, Mn-induced mitochondrial dysfunction and ROS overproduction also contributes to activation of redox-active NF-κB. Mn was also shown to activate JAK2/STAT3 pathway [83]. Both of these mechanisms may underlie Mn-induced proinflammatory cytokine overproduction. Recent studies have also clarified the particular role of NLRP3 inflammasome activation in Mn-induced neuroinflammation and pyroptosis. Activation of NLRP3 inflammasome under Mn exposure may result not only from NF-κB-induced NLRP3 expression, but also due to exosomal transport of ACS protein from other exposed cells [90]. Mn overexposure and oxidative stress provide significant damage to lysosomes with subsequent increase in membrane permeability and cathepsin B release. The latter also up-regulates NLRP3-inflammasome activation [91].

Neuroinflammation is known to be one of the leading mechanisms of Mn-induced neurotoxicity [77] (Figure 1). Astrocytes, and particularly astrocyte activation (astrogliosis), are considered as the mediator of neurotoxic and proinflammatory effect of manganese [78]. Particularly, in mixed glial cultures Mn-induced (0–100 μM Mn for 24 h) up-regulation of proinflammatory gene expression was shown to be associated with expression of astrocyte-specific genes and especially Ccl2, being indicative of the key role of astrocytes in Mn-induced neuroinflammation [79]. At the same time, Mn-induced NF-κB activation in microglia exposed to the same metal doses significantly enhanced astrocyte activation and neuroinflammatory response [80], indicative of the important role of microglia-astrocyte interplay in Mn-associated neuroinflammation. The critical role of NF-κB pathway in Mn-induced neuroinflammation was also confirmed in mouse (50 mg/kg/day p.o. for 30 days) studies with the knockout of astrocyte-specific IκB kinase 2 that prevented the neuroinflammatory reaction [81].

Activation of NF-κB may be mediated by Mn-induced IκB-α degradation in BV2 microglia [82]. Up-regulation of JAK2-STAT3 signaling in microglia may be also responsible for microglial TNF-α and IL-1β secretion in response to Mn^2+^ exposure in mice (2, 5, 10 mg/kg MnCl_2_ i.g. for 30 days) [83]. In a C57/BL mouse model (100 mg/kg Mn i.p. once in three days for two weeks) LRRK2 was also shown to play a significant role in up-regulating microglial activation and increased IL-1β and TNF-α expression, being also associated with microglial autophagy dysfunction as demonstrated by elevated Atg5 and Beclin-1 levels [84]. The observed Mn-induced (100 μM Mn for 24 h) increase in proinflammatory cytokine secretion is also dependent on mitochondrial dysfunction and down-regulation of mitofusin 2 (Mfn2), whereas mitochondrial protection with Mito-apocynin significantly ameliorated the proinflammatory effects of the metal [85].

Recent studies demonstrated that proinflammatory effect of Mn in brain tissues may be mediated by inflammasome expression and activation. Particularly, Mn exposure in rats (2, 5, 10 mg/kg MnCl_2_ i.g. for 30 days) resulted in striatal NF-κB activation leading to the formation of NLRP3 inflammasome complex and the consequent ROS-mediated activation with subsequent IL-1β and IL-18 secretion by microglia [86]. Mn-induced activation of NLRP3-CASP1 (caspase 1) inflammasome pathway in Mn-exposed rats (100 mg/kg Mn s.c. 3 times a week) and BV2 cells (100 μM Mn for 6 h) may also be associated with autophagy-lysosomal dysfunction, whereas release of lysosomal CTSB (cathepsin B) plays a significant role in Mn-induced NLRP3-CASP1 inflammasome activation [87]. In turn, PAS-Na treatment prevented Mn-induced expression of NLRP3, CASP1, IL-1β, and IL-18 in BV2 microglia cells exposed to 200 μmol/L MnCl_2_ for 24–48 h [88]. Correspondingly, para-aminosalicylic acid in Mn-exposed Sprague-Dawley rats (5, 10, 20 mg/kg Mn i.p. 5 days per week for eight weeks) was shown to reduce Mn-induced NLRP3 inflammasome dependent pyroptosis through inhibition of NF-κB signaling, that may occur due to decreased p65 and IkB-α phosphorylation and ROS production [89]. Moreover, alternative mechanism of NLRP3 inflammasome pathway activation may involve Mn-induced release and cell-to-cell transfer of inflammasome adaptor protein ASC-containing exosomes, as demonstrated in primary microglial cells (100 μM Mn for 6–24 h) [90].

Therefore, key pathways mediating neuroinflammatory effect of Mn exposure appear to involve NF-κB, JAK2/STAT3, and NLRP3 inflammasome activation (Figure 1).

### 3.6. Mitochondrial Dysfunction and Oxidative Stress

Although prooxidant effects of Mn have been reported and extensively studied through the decades, investigations performed in recent years have clarified the mechanisms underlying the impact of Mn on mitochondrial ROS generation, as well as transcriptional regulation of redox homeostasis, and its relationship to mitochondrial dysfunction [92].

**Figure 2:**
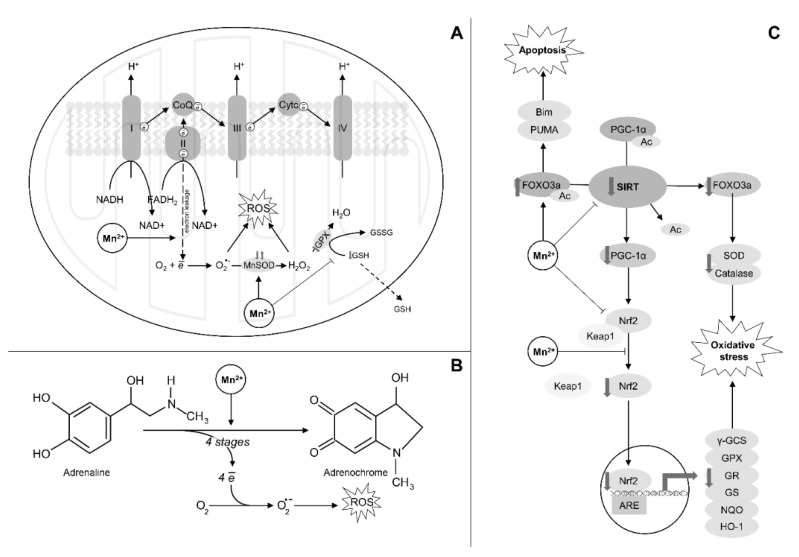
The potential mechanisms of Mn-induced oxidative stress. (**A**) Mn overexposure increases electron leakage and superoxide generation at electron transport chain complex II and increases MnSOD-dependent hydrogen peroxide formation [94,95]. Depression of antioxidant enzymes and loss of low-molecular weight antioxidants in response to Mn exposure also contribute to increased ROS accumulation [94]. (**B**) Mn increases adrenaline oxidation to adrenochrome with subsequent overproduction of superoxide [97]. (**C**) The impact of Mn on redox homeostasis may also be regulated at transcriptional level. Specifically, Mn-induced sirtuin down-regulation [98] results in increased acetylation of FOXO3a and PGC1α. Increased PGC1a acetylation is associated with reduced Nrf2 expression and down-regulation of Nrf2 target genes including γ-glutamylcysteine synthetase (γ-GCS), glutathione peroxidase (GPX), glutathione reductase (GR), glutathione synthetase (GS), NAD(P)H Quinone Dehydrogenase 1 (NQO-1), heme oxygenase 1 (HO-1) [98]. Mn exposure may also affect Nrf2 signaling through alterations of Keap1 expression [98]. In turn, increased FOXO3a acetylation results in decreased SOD and catalase expression that are up-regulated by deacetylated form, as well as promotes proapoptotic signaling through Bim and PUMA [103].

Mn-induced mitochondrial dysfunction was shown to be among the leading mechanisms of prooxidant effect of the metal (Figure 2A). Specifically, in neuronal cultures Mn exposure (1, 3, 5 mM Mn for 24–48 h) resulted in elevation of mitochondrial ROS generation [91] and reduction of mitochondrial membrane potential (MMP) [93]. Using rat heart mitochondria, it has been demonstrated that Mn^2+^ exposure (5–500 µM Mn 0–20 min) increases ROS production at respiratory chain complex II, increases superoxide dismutation, promotes the loss of low-molecular weight antioxidants via mitochondrial permeability transition pores, as well as increases ROS production by flavin-containing oxidoreductases of the Krebs cycle [94]. Generally, the role of complex II as a target for Mn toxicity corroborates our findings on different modes of prooxidant effect of Mn (0–200 µM Mn for 24 h) and rotenone, a specific electron transport chain (ETC) complex I inhibitor, in human-induced pluripotent stem cell-derived postmitotic mesencephalic dopamine neurons [95]. However, our recent findings demonstrate that mitochondrial dysfunction is observed only at cytotoxic exposure doses (0–300 µM for 24 h), being indicative that there are neurotoxic insults not associated with acute cell death that are independent of mitochondria dysfunction [96].

Mn was also shown to increase adrenaline oxidation to adrenochrome with subsequent ROS generation, oxidative DNA damage, and alteration of RNA synthesis in T7 RNA polymerase-driven transcription [97] (Figure 2B).

A significant progress was achieved in the studies of the interplay between Mn and Nrf2, being the key transcriptional regulator of antioxidant system and redox homeostasis (Figure 2C). It has been found that Mn exposure (1 μM–200 μM for 24 h or two weeks) down-regulates Nrf2 signaling through alteration of Keap1 expression altogether resulting in reduced expression of antioxidant enzymes and heat shock proteins [98]. However, the effect of Mn on the Keap1–Nrf2–ARE signaling pathway was found to be biphasic. Thus, intraperitoneal exposure to 12.5 mg/kg Mn for two weeks was found to increase Nrf2 and reduce Keap1 expression in rat striatum as compared to controls, whereas exposure to higher doses (25 and 50 mg/kg) resulted in opposite effects. Similar trend was observed for heme oxygenase (HO-1) and NAD(P)H quinone dehydrogenase 1 (NQO1) expression. However, the negative impact of Mn exposure on γ-glutamylcysteine synthetase, GPX, GST, and GR was found to be dose-dependent [99]. It has been also demonstrated that histone hypoacetylation may result in inhibition of Mn-induced Nrf2/HO-1 pathway in PC12 cells, thus promoting Mn-induced ROS generation in PC12 cells exposed to 0–300 μM Mn for 24 h [100]. An additional mechanism of Mn-induced alteration in Nrf2 signaling may involve activation of GSK-3β [101] which is known to possess modulatory effects on Nrf2 [102]. Generally, the existing data demonstrate that lower toxic Mn exposure may activate Nrf2 signaling as a compensatory response to Mn-induced oxidative stress, whereas at high-dose exposure Nrf2 will be inhibited, thus reducing resistance to Mn-induced oxidative stress and toxicity.

The impact of Mn on redox metabolism may be mediated through its interference with sirtuin (SIRT) signaling, being considered as the key regulator of antioxidant system through regulation of Nrf2 (Figure 2C). Specifically, it has been demonstrated that down-regulation of SIRT1 under Mn exposure (0–1000 μM Mn for 24 h) is associated with proapoptotic signaling and FOXO3a activation [103]. In Mn-exposed primary cultured neurons (100, 200 μM Mn for 24 h) up-regulation of SIRT3 expression was shown to be involved in protective effects of resveratrol against Mn-induced mitochondrial dysfunction [104] which may be at least partially mediated by the role of SIRT3 in regulation of Mn-SOD activity [105].

The role of Mn in regulation of redox homeostasis, especially in mitochondria, is also mediated by mitochondrial MnSOD [106]. However, data on the association between Mn exposure and MnSOD activity are highly variable. Interestingly, along with increased ROS production and depression of other antioxidant enzymes, Mn exposure in rats (100 mg/kg Mn i.p. for 7 days) significantly reduced brain mitochondrial MnSOD levels [107]. At the same time, in SH-SY5Y cells Mn exposure (0–100 μM Mn for 5 h) resulted in an increase in cellular oxygen consumption rate, SOD2 activity, and H_2_O_2_ production without a significant elevation of superoxide production observed over entire physiological to pathological range [108]. These findings contradict our observations of lack of Mn-induced mitochondrial dysfunction at exposure ranges lower than cytotoxic [96]. Given this inconsistency one could propose the physiological regulatory role of Mn-induced mitochondrial H_2_O_2_ production at nearly physiological exposure ranges. A detailed in vitro study demonstrated 500 μM Mn-induced up-regulation of MnSOD mRNA and protein levels that was found to be dependent on protein tyrosine kinase (PTK) or protein kinase C (PKC) signaling [109]. The association between systemic Mn levels and MnSOD is still unclear.

### 3.7. Endoplasmic Reticulum Stress

A role of Mn exposure in endoplasmic reticulum stress and its contribution to apoptosis and neurotoxicity has been demonstrated earlier [110]. Recent studies have confirmed earlier observations and highlighted additional mechanisms underlying this association. Mn exposure (5–30 mg/kg Mn i.p. for 3–4 weeks) induced a dose-dependent increase in CHOP, GRP78, and caspase 12 [111], GADD34, XBP-1 [112], ATF-6α, PERK, Sigma-1R, as well as proapoptotic protein expression in rat striatum [113]. It has also been demonstrated that Mn-induced α-synuclein accumulation and toxicity may be mediated through ERS and apoptosis [114].

At the same time, a recent study demonstrated that the impact of Mn on endoplasmic reticulum may differentially modulate apoptosis. Although prolonged ERS due to Mn exposure is shown to up-regulate apoptosis, unfolded protein response following Mn exposure in SH-SY5Y cells (0–100 μM for 24 h) induced autophagy as a protective response to metal toxicity through inositol requiring enzyme 1 (IRE1) signaling. The latter was shown to stimulate ASK1-TRAF2 complex formation with subsequent JNK activation and Beclin-1 mRNA expression [115]. Another mechanism of ERS-associated autophagy may include activation of PERK/eIF2α/ATF4 signaling pathway as demonstrated in SH-SY5Y cells exposed to 100 μM Mn for 6–24 h [116]. ERS-induced autophagy was also shown to be protective against Mn-induced α-synuclein oligomerization [117].

### 3.8. Autophagy

Autophagy is considered as a compensatory response to Mn toxicity in neuronal cells [118], whereas dysregulation of autophagy is considered as the potential mechanism linking perturbations in Mn metabolism and neurodegeneration [119].

The impact of Mn exposure on autophagy was shown to be time dependent in BV-2 microglial cells with complete functional autophagy of cellular compartments damaged by Mn toxicity at low-to-moderate Mn exposure (250–750 μM Mn for 24 h). In contrast, the high rate of Mn-induced damage results in lysosomal membrane permeabilization, cathepsin release, and dysregulated autophagy, altogether leading to cell death [120]. In parallel with lysosomal membrane permeabilization, another mechanism of Mn-induced regulated necrosis revealed at similar exposure doses in microglia involves complex events including DNA damage, AIF nuclear translocation, mitochondrial membrane permeabilization, and poly (ADP-ribose) polymerase 1 (PARP1)-dependent cell death, altogether referring to “parathanatos” [121]. Oppositely, in a range of 6.25–100 μM Mn was shown to induce autophagic flux in Huntington’s disease cell models resulting in autophagic sequestration of huntingtin (Htt) aggregates, thus possessing protective effect [122].

The observed effects of different Mn exposure times on autophagy [120] corroborate earlier data demonstrating time-dependent S-nitrosylation of the key proteins being involved in autophagy. Specifically, long-term Mn exposure (400 μM Mn for 24 h) up-regulated inducible NOS activity and NO production with subsequent JNK and Bcl2 S-nitrosylation resulting in autophagy inhibition [123]. A later study by this research group demonstrated that IKKβ S-nitrosylation may also affect autophagy through reduction of AMPK phosphorylation and subsequent mTOR pathway activation [124]. Dysregulation of autophagy in response to Mn exposure (200μM Mn for 0–100) may be at least partially mediated by reduced nuclear localization and activity of TFEB, a key regulator of autophagy, thus leading to the accumulation of dysfunctional mitochondria [125]. Mn-induced α-synuclein overproduction was also shown to disrupt HMGB1-dependent autophagy affecting HMGB1-Beclin1 interaction and promoting Beclin1-Bcl2 binding in exposed SH-SY5Y cells (50–200 μM for 24 h) [126]. Up-regulation of autophagy is considered as a potential protective mechanism of spermine against Mn-induced degeneration of dopaminergic neurons exposed to 300 and 600 μM for 24 h [127].

Recent studies demonstrated that mitophagy may occur as the particular mechanism of Mn-induced autophagy [118]. Specifically, Mn exposure (250 μM MnCl_2_ for 2–24 h) resulted in ROS-dependent mitochondrial dysfunction and subsequent mitophagy as evidenced by increased LC3-II/LC3-I, Beclin-1, PINK1, and P-parkin expression. Increased nuclear FOXO3 translocation under Mn treatment and reduced mitochondrial autophagy in FOXO3 KO cells demonstrate that Mn-induced mitophagy may be at least partially mediated by FOXO3 signaling [128]. Correspondingly PINK1/Parkin-mediated mitophagy was shown to be essential for apoptotic resistance under Mn exposure (250–2000 μM for 24–48 h) in dopaminergic neuronal cells [129].

### 3.9. Arginase

Ureohydrolases arginase and agmatinase are Mn-dependent enzymes containing two Mn^2+^ atoms in the active center, although only one of them promotes catalysis and another one enhances enzyme activity [130]. A recent study proposed that active site Mn^2+^ cation is not directly involved in the charge-transfer process during reaction, being involved in stabilization of the nucleophile and intermediates [131].

Recent studies demonstrated the role of arginase as a physiological target of Mn in a number of pathologies. Specifically, overexpression of arginase I, but not arginase II, may possess neuroprotective effects in cortical injury through reducing contusion volume, abnormal neuronal morphology, and improvement in NO metabolism [132]. In a model of Huntington’s disease, altered urea cycle and the underlying decrease in arginase II activity, but not its expression, was noted, indicative of reduced bioavailable Mn. In turn, Mn supplementation (50 mg/kg s.c. at days 0, 3, and 6) resulted in an increased enzyme activity thus supporting the association between Mn deficiency and striatal pathology [133]. These findings, although being relatively sparse, may be indicative of the physiological role of Mn in neuronal health that may be observed only at physiological levels.

### 3.10. Apoptosis

**Figure 3:**
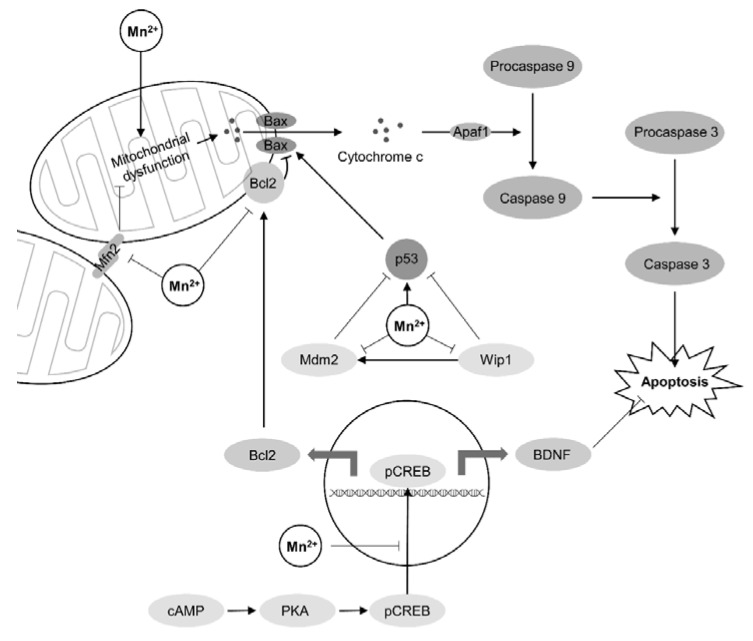
Mechanisms of Mn-induced apoptosis. Mn exposure results in mitochondrial dysfunction and Bax-associated cytochrome c leakage with subsequent caspase 9 and 3 activation resulting in apoptosis. Mn-induced apoptosis may be aggravated by stimulatory effects of manganese on p53 protein as well as down-regulation of murine double minute 2 (Mdm2) homolog and wild-type p53-induced phosphatase 1 (Wip1) protein, both having inhibitory influence on p53 [140]. The impact of Mn on p53 signaling may be also mediated by Mn-dependent modulation of ataxia telangiectasia mutated (ATM) kinase [71]. In turn, Mn may decrease anti-apoptotic effects of Bcl2 and BDNF through inhibiting CREB phosphorylation and subsequent down-regulation of Bcl2 and BDNF expression [137]. It has been also demonstrated that mitochondrial pathway of apoptosis may be also aggravated by Mn-induced alteration of mitofusin 2 (Mfn2) expression, a protein involved in mitochondrial fusion and functioning [134].

Apoptosis is considered as one of the key cellular events underlying Mn-induced neurodegeneration. The most recent research in the field revealed the intimate mechanisms of Mn-associated proapoptotic signaling (Figure 3). In addition to the clearly demonstrated activation of caspase 3 following mitochondrial dysfunction and cytochrome c leakage, it has been observed that alteration of mitofusin 2 (Mfn2) expression in rat striatum and PC12 cells may also contribute to caspase 3 activation upon Mn exposure (2–25 mg/kg Mn i.p. for 30 days) [134]. Mn-induced ROS overproduction was shown to activate MEK/ERK5 signaling pathway resulting in ERK5-dependent Bcl-2 phosphorylation with subsequent inhibition, thus promoting proapoptotic signaling in MN9D cells (200–2000 μM for 24–48 h) [135] (Figure 3). In turn, Mn-induced apoptosis was shown to mediate alterations in spatial learning and memory deficits in metal-exposed animals (30 mg/kg Mn p.o. for 35 days) [136].

Alteration of cAMP/PKA/MAPK/CREB pathway was also shown to play a significant role in Mn-induced apoptosis in PC12 cell line (0–600 μM Mn for 24 h) through down-regulation of BDNF expression and Bcl-2 levels [137]. This pathway may be also modulated by alterations of intracellular Ca^2+^ levels and subsequent increase in MAPK and CREB phosphorylation in response to an increase in Ca^2+^ levels induced by Mn exposure in PC12 cells (0–500 μM for 0–24 h) [138]. Impaired Ras/MAPK and PI3/Akt signaling in cortical neurons following Mn exposure (0–400 μM for 4–24 h) may be mediated by interruption of NT3/TrkC signaling altogether being associated with apoptosis, whereas treatment with hNT3 ameliorated Mn-induced proapoptotic events, thus being indicative of the role of NT3/TrkC pathway in Mn neurotoxicity and apoptosis [139].

Recent studies have unraveled multiple regulators that could be considered as candidate targets for Mn-induced neuronal apoptosis. Specifically, p53 activation in Mn-exposed cells (0–1000 μM for 24 h) was found to be associated with downregulation of wild-type p53-induced phosphatase 1 (Wip1) protein expression and a subsequent inhibition of murine double minute 2 (Mdm2) homolog in rat striatum [140]. Mn exposure (300 μM Mn for 6–24) was also shown to depress p73 mRNA expression in an N27 dopaminergic neuronal model thus increasing susceptibility of neuronal cells to apoptosis [141]. Mn-induced apoptosis may be at least partially dependent on K-homology splicing regulator protein (KHSRP) up-regulation that was found to be overexpressed in association with proapoptotic genes and colocalized with active caspase-3 in PC12 cells exposed to Mn (0–1000 μM for 1–24 h) [142].

## 4. Neurodegeneration

In parallel with epidemiological studies demonstrating the association between Mn exposure levels and neurodegeneration, recent studies have deepened the understanding of the interference between Mn exposure and amyloid β, tau protein, and α-synuclein accumulation. Mn has been implicated in the etiology of several neurodegenerative disorders, which will be discussed below.

### 4.1. Amyloid β and Tau

Mn exposure was found to be a risk factor for Alzheimer’s disease through up-regulation of Amyloid β accumulation [143]. However, the existing data on the impact of Mn exposure on amyloidogenesis are still insufficient. Despite evidence on direct interaction between Mn^2+^ ion and amyloid, metal binding to Aβ_1-40_ N-terminal part was found to be weak and unlikely to have significant effect on protein aggregation into amyloid fibrils [144], being indicative of the role of Mn-induced modulation of amyloidogenesis rather than direct Mn-Aβ interaction in amyloid pathology. Specifically, a recent study demonstrated that Mn exposure (0–500 μM MnCl_2_ for 24 h) increased Aβ_1-42_ secretion through up-regulation of β-secretase 1 (BACE1), APP, and presenilin (PS1) expression in APPsw-N2a cells only in the presence of microglia or microglia-conditioned medium. Mn-induced microglia activation with IL-1β and TNFα secretion may further aggravate the process [145]. Correspondingly, Mn exposure in SN56 basal forebrain cholinergic neurons (25–300 for 24h and 14 days) resulted in a significant increase in Aβ and tau protein accumulation that may be mediated by heat shock protein and proteasome dysfunction [146]. In addition, L-NAME pretreatment significantly increased the protective effects of naringerin upon Aβ_1-B_ and Mn^2+^ exposure in rats (0.8 mg/kg Mn intranasal for 21 days), indicative of a role for iNOS in the pathogenesis of Mn and amyloid beta neurotoxicity [147].

Tau, another Alzheimer’s disease-related protein, was also affected by Mn exposure. Specifically, accumulation of hyperphosphorylated tau under Mn exposure (500–1000 μM for 24 h) was also shown to be associated with demethylation of protein phosphatase 2 A (PP2A) that is known to be one of the key tau phosphatases [148]. Correspondingly, reversal of PP2A demethylation was associated with reduction of pTau levels, reduced oxidative stress, apoptosis, and improvement in cell viability [149]. In addition, Mn nanoparticles were shown to induce a shift to a more packed tau structure associated with proapoptotic signaling as evidenced by caspase-3 and caspase-9 activation, as well as Bax/Bcl-2 ratio elevation in the exposed SH-SY5Y cells (1–200μg/mL Mn for 24 h) [150].

### 4.2. Synuclein

Although earlier studies demonstrated poor affinity of α-synuclein (α-syn) for Mn^2+^ [151], a recent study revealed potential binding sites and a shift to a more compacted α-syn structure upon Mn binding which may also affect protein folding and its cytotoxic properties [152]. In addition, in brain slices exposed to 400 μM Mn for 24 h, α-syn oligomerization was shown to be calpain-1-dependent [153]. In turn, a recent study demonstrated that α-syn overexpression results in increased cellular Mn release without altering metal transporter genes, indicative of the role of α-syn in Mn storage [154].

In parallel with data on direct interaction between Mn and α-syn, recent findings demonstrated the role of Mn exposure in aggravation of α-syn neurotoxicity. Mn (100 µM for 24 h) exposure-induced increase in α-syn expression was also found to interact with TrkB receptors, inhibit BDNF/TrkB signaling, and affect Fyn-mediated phosphorylation of GluN2B subunit thus resulting in impaired NMDAR signaling [155]. Mn-induced modulation of GABA receptors with up-regulation of GABABR and down-regulation of GABAAR may also contribute to α-syn accumulation with subsequent down-regulation of CREB signaling and BDNF levels as demonstrated in Mn-exposed rats (6.55 mg/kg Mn for 4–12 weeks) and SH-SY5Y neuroblastoma cells (250–1000 μM for 24 h) [156].

Mn also potentiates neuroinflammatory effects of α-syn through a shift to the proinflammatory M1 microglial phenotype characterized by proinflammatory molecules overexpression (IL-6, IL-12b, IFN-β, IL-1α, and IL-1β CXCL2, CXCL3, CXCL10, CCL5-R, and Nos2), as well as NLRP3 inflammasome activation [157]. α-syn was also shown to be involved in dysregulation of Mn-induced autophagy, thus promoting neuronal injury in exposed α-Syn gene knockout and wild-type mice (50–200 μmol/kg i.p. 5 days/week for six weeks) [158]. It is also notable that Atp13a2 deficiency increases susceptibility to Mn overload (5 mg/kg i.p. for 30 days) resulting in increased brain Mn and insoluble α-syn accumulation [159].

Significant progress was achieved in understanding the role of exosomes in interactive neurotoxic effects of Mn and α-syn. Specifically, Mn exposure (300 μM MnCl_2_ for 24 h) in α-syn-expressing cells increased expression of Rab27a protein, thus promoting the release of exosomes containing miRNA that are involved in regulation of protein aggregation, autophagy, inflammation, and hypoxia [160]. Correspondingly, in cultured dopaminergic neuronal cells Mn exposure (300 μM Mn for 24 h) resulted not only in misfolded α-synuclein accumulation, but also induced secretion of α-syn-containing exosomes into the extracellular medium with their subsequent microglial endocytosis and propagation of neuroinflammatory response [161].

Another mechanism of the interaction between Mn exposure and α-syn may involve modulation of miRNAs expression. Specifically, in human neuroblastoma SH-SY5Y Mn exposure (100 μM for 24 h) resulted in a significant reduction in miR-7 and miR-433 expression with subsequent increase in molecular targets α-syn and fibroblast growth factor 20 mRNA expression [162].

However, the effects of α-syn in terms of Mn neurotoxicity were found to be non-linear. Particularly, physiological α-syn expression was significantly reduced Mn-induced neuronal apoptosis through down-regulation of cytochrome c release, caspase 3 and 9 activity, pro-apoptotic PKCδ activation, although prolonged Mn exposure (300 μM for 24 h) resulted in α-syn overexpression and aggregation [163]. These findings generally corroborate our earlier data on the protective effects of α-Syn against Mn accumulation and oxidative stress in *C. elegans* exposed to 0–10 mM Mn [164].

## 5. Neurotransmission

Recent studies have demonstrated a significant impact of Mn exposure in mice (25–100 μmol/kg i.p. for 24 days) on synaptic vesicle fusion through alteration of SNARE complex formation through calpain-dependent SNAP-25 cleavage [165]. It has been also demonstrated in SH-SY5Y cells exposed to 0–200 μM Mn for 0–24 h, that Mn-induced α-syn overexpression may also contribute to this mechanism [166]. In addition, Mn exposure (100 μM for 0–24 h) may be also responsible for down-regulation of SNAP-25 expression as well as impairment of SNARE-associated protein interaction in cultured neurons [167]. Impairment of synaptic vesicle fusion under Mn exposure (100 μM Mn for 24h) was shown to be mediated by the interference of α-syn accumulation with synaptotagmin/Rab3-GAP and Rab3A-GTP/Rab3-GAP signaling [168]. Taken together, these mechanisms may underlie Mn-induced alterations of neurotransmitter release. At the same time, recent studies have also unraveled the interference between Mn exposure and neurotransmitter metabolism [28].

### 5.1. Glutamate

**Figure 4:**
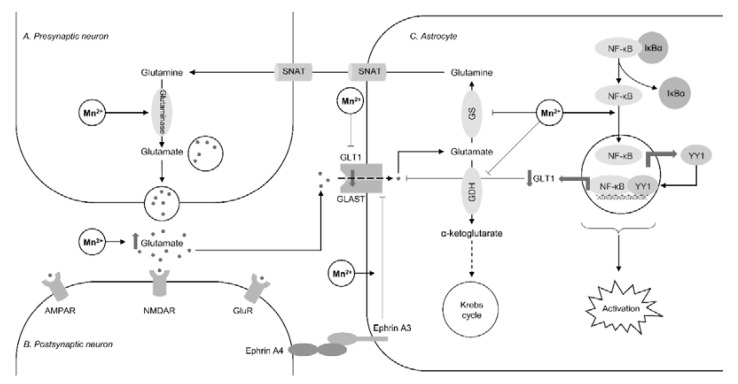
The impact of manganese overexposure on glutamate-glutamine cycle. Manganese exposure results in a significant increase in glutamate levels through down-regulation of glutamine synthetase (GS) [179] and glutamate dehydrogenase (GDH) [182] along with up-regulation of glutaminase [179]. These effects result in reduced glutamate-to-glutamine conversion as well as glutamate catabolism in Krebs cycle through the formation of α-ketoglutarate. Mn-induced inhibition of astrocyte glutamate uptake results from inhibition of glutamine transporters (GLT1 and GLAST). Recent studies demonstrated that this inhibitory effect may be mediated through NF-κB-dependent activation of Yin Yang 1 (YY1) transcription factor [173] and ephrin A3 [178]. It is also notable that Mn-induced NF-κB signaling also plays a significant role in astrocyte activation associated with reduced glutamine synthetase activity [181].

Regulation of glutamate transporters by Mn is considered as one of the key mechanisms of Mn impact on glutamatergic system [169] (Figure 4). Recent studies have confirmed earlier data and generated new data on Mn-dependent transcriptional epigenetics and translational regulation of glutamate transporters [170]. Mn exposure (30 mg/kg intranasal for 21 days) was shown to decrease EAAT1 (GLAST) and EAAT2 (GLT-1) promotor activities, as well as mRNA and protein levels resulting in reduced glutamate uptake in human astrocyte H4 cells as well as murine brain, being associated with neurobehavioral deficiency, reduced tyrosine hydroxylase mRNA and protein levels, as well as reduced histone H3 and H4 acetylation [171], all being reversed by valproic acid and sodium butyrate [172]. Our previous data from both in vitro (exposure to 250 μM Mn for 6 h) [173,174] and in vivo (exposure to 30 mg/kg intranasal for 21 days) [175] studies demonstrate that activation of Yin Yang 1 (YY1) transcription factor plays a significant role in GLAST and GLT-1 expression, whereas YY1 knockout significantly attenuated Mn-induced motor dysfunction, glutamate transporter reduction, tyrosine hydroxylase activity, as well as histone deacetylation [176]. At the same time, it has been demonstrated that a time- and dose-dependent increase in GLAST activity in chick cerebellar Bergmann glia cells in response to acute but non-toxic Mn exposure (200 µM for 30 min) may be associated with increased transporter affinity for [^3^H]-d-aspartate [177]. Using fluoxetine as an ephrin-A3 inhibitor it has been demonstrated that alterations in glutamate transporters and metabolism in Mn-exposed Kinming mice (50 mg/kg MnCl_2_ s.c. for two weeks) and cultured primary astrocytes (500 μM for 24 h) may be ephrin-A3-dependent [178].

Mn-induced decrease in GLAST and GLT-1 mRNA expression along with inhibition of glutamine synthetase and up-regulation of phosphate-activated glutaminase resulted in a significant increase of Glu and decline in Gln levels in hippocampus, thalamus, striatum, and globus pallidus of Mn-exposed (15 mg/kg i.p. for four weeks) rats [179]. However, an in vitro study using brain-derived mitochondrial fractions demonstrated that Mn is capable of inhibiting phosphate-activated glutaminase with IC50 = 2317 μM in a dose-dependent manner analogous to ammonia [180]. Inhibition of astrocytic glutamine synthetase in response to 100 µM exposure for 24 h may be mediated by microglial activation and its impact on astrocyte reactivity [181]. Another enzyme of glutamate catabolism, glutamate dehydrogenase, was found to be inhibited by Mn^2+^ exposure with the second isoenzyme (GDH2) showing greater sensitivity to metal-induced inhibition as compared to GDH1 [182].

Mn exposure (0–30 mg/kg i.p. for three weeks) resulted in a significant decrease of hippocampal mRNA and protein expression of NMDA receptor subunits (NR1, NR2A, and NR2B), as well as CREB and BDNF in newborn Sprague–Dawley rats [183].

### 5.2. γ-Aminobutyric Acid (GABA)

In welders, thalamic GABA levels were found to be associated with Mn deposition and the rate of Mn exposure, being the highest values observed at high exposure with air Mn levels of 0.23 ± 0.18 mg/m^3^ [184]. Specifically, thalamic GABA levels in welders significantly correlated with air Mn concentrations and cumulative exposure for the last three and 12 months [185]. However, another study did not observe any association between thalamic and striatal GABA levels and blood Mn or airborne metal exposure levels in welders [186]. This inconsistency may be explained by non-linear association between Mn exposure and brain GABA alterations, with the latter being undetectable at lower doses and periods of exposure [187].

Serum GABA levels were found to be reduced in association with circulating Gln and thyroid hormone levels in response to subacute Mn exposure (7.5–30 mg/kg i.p. five days/week for four weeks) in rats [188]. Another study demonstrated that Mn exposure (6.55 mg/kg Mn i.p. five days/week for 12 weeks) significantly decreased basal ganglia GABA levels, glutamate-to-GABA ratio, as well as affected GAD and GABA-T activity, and GAT-1 and GABAA mRNA expression in rats [189,190]. Reduced hypothalamic GABAA receptor subunits protein expression in response to Mn exposure (2.5–10 mg/kg oral for 11 days) was associated with increasing NO production through amelioration of inhibitory effect of GABA on NO synthase, altogether leading to aberrant gonadotropin-releasing hormone (GnRH) production [191].

### 5.3. Dopamine

Manganese is known to interfere with dopamine signaling being the leading pathogenetic mechanism of Parkinson’s disease [192]. Dopaminergic neurons are considered as one of the main targets for Mn toxicity with predominant accumulation of the metal in substantia nigra pars reticulata and pars compacta and its localization adjacent to the nucleus [193]. In turn, Mn-induced dopaminergic neuron loss associated with motor activity deficits in rats intraperitoneally injected with Mn (1–5 mg/kg every 10 days for 150 days) [194]. At the same time, our earlier study in *C. elegans* demonstrated that Mn exposure (15–45 mM for 4 h) resulted in a significant decline in dopamine levels without loss of dopaminergic neurons [195]. A study using Mn-exposed (5–6.7 mg/kg Mn for 25–80 weeks) non-human primates demonstrated a significant decrease in dopamine release in the frontal cortex in 4 of 6 animals [196].

Interference between dopamine signaling and Mn may be mediated by its influence on tyrosine hydroxylase. Specifically, Mn exposure (10 mg/kg/day i.g. for 30 days) intensified striatal dopamine and nigral tyrosine hydroxylase loss in MitoPark mouse. These alterations were also associated with Mn-induced mitochondrial dysfunction, oxidative stress, microglia activation and neuroinflammation, as well as PD-associated protein oligomerization [197].

However, the impact of Mn exposure on tyrosine hydroxylase and dopamine metabolism was found to be not similar through a lifespan. Specifically, shortly after early-life Mn exposure (5–20 mg/kg i.p. for four days) in rats a significant increase in striatal TH protein levels and TH phosphorylation at Ser19, Ser31, and Ser40 was observed. At the same time, in adulthood TH levels were found to be reduced in a dose-dependent manner in association with increased phosphorylation at Ser19 and Ser40 [198]. Nearly similar age-dependent effect on TH activity was observed in zebrafish larvae exposed to 0.1–0.5 mM for five days [199].

Certain studies have highlighted the mechanisms of transcriptional regulation of tyrosine hydroxylase under Mn exposure. Transcription factor RE1-silencing transcription factor (REST) was shown to overwhelm Mn-induced alterations in TH activity through up-regulation of mRNA and protein transcription in dopaminergic neuronal cells, as well as ameliorated other toxic effects of Mn exposure (250 μM for 12 h) including apoptosis, inflammation, and oxidative stress [120]. Kumasaka et al. (2017) demonstrated that a decline TH expression in TGW cells may be mediated by Mn (30–100 μM for 24 h) exposure-induced down-regulation of mRNA and protein transcription, as well as increased degradation of c-RET kinase [200,201].

In addition, in vivo Mn exposure (0–50 mg/kg i.p. for 2 weeks) was shown to cause reduction in striatal dopamine D1 receptor and N-methyl-D-aspartate receptor subunits (NR1 and NR2) mRNA and protein expression, as well as inhibition of DR1 and NMDAR interaction, being associated with altered spatial learning and memory in mice [202]. Examination of workers with clinical parkinsonism exposed to 2.6 mg Mn/m^3^-years revealed increased nigral D2R non-displaceable binding potential, being also associated with duration of occupational Mn exposure and motor dysfunction [203].

### 5.4. Catecholamines

Catecholaminergic neurotransmission was also found to be affected by Mn exposure. In parallel with markers of dopaminergic dysfunction, Mn exposure in rats (0–50 mg/kg i.g. for 21–100 days) resulted in a significant decrease in norepinephrine levels, evoked norepinephrine release, resulting in medial prefrontal cortex catecholaminergic dysfunction and being associated with impaired attention, motor dysfunction, and altered impulse control [204]. Reversal of Mn-induced alterations of motor functions with methylphenidate treatment in Mn-exposed (0–50 mg/kg i.g. for 145 days) rats underlines the role of prefrontal cortex and striatal catecholaminergic dysfunction in Mn-associated motor impairment [205]. Correspondingly, although Mn exposure (0–50 mg/kg i.g. for 21–500 days) resulted in a significant reduction of potassium-stimulated extracellular norepinephrine, dopamine, and serotonin levels, as well as striatal dopamine levels, the observed alterations of attention and fine motor function are indicative of the role of Mn-induced catecholaminergic dysfunction in neurobehavioral disorders [206].

### 5.5. Acetylcholine

Mn exposure (25–300 for 24 h and 14 days) was shown to disrupt cholinergic neurotransmission in basal forebrain cholinergic neurons through up-regulation of AChE mRNA expression and protein activity in parallel with inhibition of cholineacetyltransferase activity and down-regulation of high-affinity choline transporter mRNA, as well as cholinergic neuron death, altogether resulting in reduced acetylcholine levels [146].

The observed increase in hypothalamic, cerebral, and cerebellar AChE activity in Mn-treated (15 mg/kg i.g. for 45 days) rats was also associated with increased ROS production and depletion of the antioxidant system in these brain regions, as well as locomotor and motor deficits [207]. These findings are in agreement with the observation of Mn (30 mg/kg Mn p.o. for 35 days) exposure-induced increase in striatal and hippocampal acetylcholinesterase activity in rats and its reversal by rutin treatment [136].

Mn exposure (10–50 mM for 30 min) during L1 larval stage in *C. elegans* also resulted in a significant increase in AChE mRNA expression as well as dose-dependent cholinergic neurodegeneration, both being aggravated when co-exposed to Mn and methylmercury (MeHg) [208].

## 6. Conclusions

Recent findings have shed light and broadened our understanding on the mechanisms associated with the earlier observed neurotoxic effects of Mn. Significant progress was achieved in understanding the role of Mn transporters SLC39A14 (ZIP14), SLC39A8 (ZIP8), SLC30A10 (ZNT10) in regulation of systemic and brain manganese handling. Genetic analysis identified multiple metabolic pathways that could be considered targets for Mn neurotoxicity, although these pathways may be also affected by epigenetic effects of Mn exposure. Corroborating earlier data as well as (epi) genomic and metabolomic profiling, the key mechanisms involved in Mn neurotoxicity include oxidative stress, endoplasmic reticulum stress, apoptosis, neuroinflammation, and interference with neurotransmitter metabolism, to name a few. However, recent findings have demonstrated the impact of Mn exposure on transcriptional regulation of these pathways such as those inherent to oxidative stress via Mn-induced modulation of sirtuin and Keap1-Nrf2 signaling. A significant role of autophagy as a protective mechanism against Mn neurotoxicity at the crossroad of mitochondrial dysfunction, endoplasmic reticulum stress, and apoptosis was also demonstrated. The impact of Mn exposure on supramolecular complexes SNARE and NLRP3 inflammasome significantly contributes to Mn-induced synaptic dysfunction and neuroinflammation, respectively. The abovementioned effects may be at least partially mediated by the impact of Mn on α-syn accumulation. In addition to Mn-induced synaptic dysfunction, impaired neurotransmission is shown to be mediated by the effects of Mn on neurotransmitter systems and their complex interplay. Although recent findings demonstrated the potential targets for Mn neurotoxicity, multiple in vitro studies have investigated the effects of Mn at concentrations far exceeding the physiologically-relevant range of 60.1–158.4 µM Mn that is known to correspond to brain levels under Mn overexposure [209]. Similarly, certain in vivo studies also used animal models exposed to physiologically and environmentally irrelevant Mn doses. Therefore, despite multiple novel mechanisms have been highlighted, additional studies are required to identify the critical targets of Mn-induced neurotoxicity and testify to their relevance to human diseases.

## References

[1] Pfalzer A.C., Bowman A.B. (2017). Relationships between Essential Manganese Biology and Manganese Toxicity in Neurological Disease. Curr. Environ. Health Rep..

[2] Chen P., Bornhorst J., Aschner M. (2018). Manganese metabolism in humans. Front. Biosci..

[3] Aguirre J.D., Culotta V.C. (2012). Battles with iron: Manganese in oxidative stress protection. J. Biol. Chem..

[4] Martinez-Finley E.J., Chakraborty S., Aschner M., Kretsinger R.H., Uversky V.N., Permyakov E.A. (2013). Manganese in Biological Systems. Encyclopedia of Metalloproteins.

[5] Michalke B., Fernsebner K. (2014). New insights into manganese toxicity and speciation. J. Trace Elem. Med. Biol..

[6] Horning K.J., Caito S.W., Tipps K.G., Bowman A.B., Aschner M. (2015). Manganese Is Essential for Neuronal Health. Annu. Rev. Nutr..

[7] Erikson K.M., Aschner M. (2019). Manganese: Its Role in Disease and Health. Met. Ions Life Sci..

[8] Martins A.C., Krum B.N., Queirós L., Tinkov A.A., Skalny A.V., Bowman A.B., Aschner M. (2020). Manganese in the Diet: Bioaccessibility, Adequate Intake, and Neurotoxicological Effects. J. Agric. Food Chem..

[9] Miah M.R., Ijomone O.M., Okoh C., Ijomone O.K., Akingbade G.T., Ke T., Krum B., da Cunha Martins A., Akinyemi A., Aranoff N. (2020). The effects of manganese overexposure on brain health. Neurochem. Int..

[10] Bowler R.M., Kornblith E.S., Gocheva V.V., Colledge M.A., Bollweg G., Kim Y., Beseler C.L., Wright C.W., Adams S.W., Lobdell D.T. (2015). Environmental exposure to manganese in air: Associations with cognitive functions. Neurotoxicology.

[11] Haynes E.N., Sucharew H., Hilbert T.J., Kuhnell P., Spencer A., Newman N.C., Burns R., Wright R., Parsons P.J., Dietrich K.N. (2018). Impact of air manganese on child neurodevelopment in East Liverpool, Ohio. Neurotoxicology.

[12] Kornblith E.S., Casey S.L., Lobdell D.T., Colledge M.A., Bowler R.M. (2018). Environmental exposure to manganese in air: Tremor, motor and cognitive symptom profiles. Neurotoxicology.

[13] Carvalho C.F.D., Oulhote Y., Martorelli M., Carvalho C.O.D., Menezes-Filho J.A., Argollo N., Abreu N. (2018). Environmental manganese exposure and associations with memory, executive functions, and hyperactivity in Brazilian children. Neurotoxicology.

[14] Siokas V., Aloizou A.M., Pateraki G., Liampas I., Mitsias P.D., Bogdanos D.P., Dardiotis E. (2021). Toxicological Risk Assessment and Multi-System Health Impacts from Exposure. https://www.elsevier.com/books/toxicological-risk-assessment-and-multi-system-health-impacts-from-exposure/tsatsakis/978-0-323-85215-9.

[15] Bowman A.B., Kwakye G.F., Herrero Hernández E., Aschner M. (2011). Role of manganese in neurodegenerative diseases. J. Trace Elem. Med. Biol..

[16] Du K., Liu M.Y., Pan Y.Z., Zhong X., Wei M.J. (2018). Association of circulating manganese levels with Parkinson’s disease: A meta-analysis. Neurosci. Lett..

[17] Roos P.M., Lierhagen S., Flaten T.P., Syversen T., Vesterberg O., Nordberg M. (2012). Manganese in cerebrospinal fluid and blood plasma of patients with amyotrophic lateral sclerosis. Exp. Biol. Med..

[18] Du K., Liu M., Pan Y., Zhong X., Wei M. (2017). Association of Serum Manganese Levels with Alzheimer’s Disease and Mild Cognitive Impairment: A Systematic Review and Meta-Analysis. Nutrients.

[19] Lucchini R., Placidi D., Cagna G., Fedrighi C., Oppini M., Peli M., Zoni S. (2017). Manganese and Developmental Neurotoxicity. Adv. Neurobiol..

[20] Liu W., Xin Y., Li Q., Shang Y., Ping Z., Min J., Cahill C.M., Rogers J.T., Wang F. (2020). Biomarkers of environmental manganese exposure and associations with childhood neurodevelopment: A systematic review and meta-analysis. Environ. Health.

[21] Balachandran R.C., Mukhopadhyay S., McBride D., Veevers J., Harrison F.E., Aschner M., Haynes E.N., Bowman A.B. (2020). Brain manganese and the balance between essential roles and neurotoxicity. J. Biol. Chem..

[22] Chung S.E., Cheong H.K., Ha E.H., Kim B.N., Ha M., Kim Y., Hong Y.C., Park H., Oh S.Y. (2015). Maternal Blood Manganese and Early Neurodevelopment: The Mothers and Children’s Environmental Health (MOCEH) Study. Environ. Health Perspect..

[23] Shih J.H., Zeng B.Y., Lin P.Y., Chen T.Y., Chen Y.W., Wu C.K., Tseng P.T., Wu M.K. (2018). Association between peripheral manganese levels and attention-deficit/hyperactivity disorder: A preliminary meta-analysis. Neuropsychiatr. Dis. Treat..

[24] Ijomone O.M., Aluko O.M., Okoh C.O., Martins A.C., Aschner M. (2019). Role for calcium signaling in manganese neurotoxicity. J. Trace Elem. Med. Biol..

[25] Martinez-Finley E.J., Gavin C.E., Aschner M., Gunter T.E. (2013). Manganese neurotoxicity and the role of reactive oxygen species. Free Radic. Biol. Med..

[26] Harischandra D.S., Ghaisas S., Zenitsky G., Jin H., Kanthasamy A., Anantharam V., Kanthasamy A.G. (2019). Manganese-Induced Neurotoxicity: New Insights Into the Triad of Protein Misfolding, Mitochondrial Impairment, and Neuroinflammation. Front. Neurosci..

[27] Wallace D.R., Taalab Y.M., Heinze S., Tariba Lovaković B., Pizent A., Renieri E., Tsatsakis A., Farooqi A.A., Javorac D., Andjelkovic M. (2020). Toxic-Metal-Induced Alteration in miRNA Expression Profile as a Proposed Mechanism for Disease Development. Cells.

[28] Soares A., Silva A.C., Tinkov A.A., Khan H., Santamaría A., Skalnaya M.G., Skalny A.V., Tsatsakis A., Bowman A.B., Aschner M. (2020). The impact of manganese on neurotransmitter systems. J. Trace Elem. Med. Biol..

[29] Nica D.V., Draghici G.A., Andrica F.M., Popescu S., Coricovac D.E., Dehelean C.A., Gergen I.I., Kovatsi L., Coleman M.D., Tsatsakis A. (2019). Short-term effects of very low dose cadmium feeding on copper, manganese and iron homeostasis: A gastropod perspective. Environ. Toxicol. Pharmacol..

[30] O’Neal S.L., Zheng W. (2015). Manganese Toxicity Upon Overexposure: A Decade in Review. Curr. Environ. Health Rep..

[31] Lucchini R.G., Aschner M., Landrigan P.J., Cranmer J.M. (2018). Neurotoxicity of manganese: Indications for future research and public health intervention from the Manganese 2016 conference. Neurotoxicology.

[32] Li J., Cen Y., Li Y. (2019). The research advances in the mechanism of manganese-induced neurotoxicity. Toxin Rev..

[33] Chen P., Chakraborty S., Mukhopadhyay S., Lee E., Paoliello M.M., Bowman A.B., Aschner M. (2015). Manganese homeostasis in the nervous system. J. Neurochem..

[34] Aydemir T.B., Kim M.H., Kim J., Colon-Perez L.M., Banan G., Mareci T.H., Febo M., Cousins R.J. (2017). Metal Transporter Zip14 (Slc39a14) Deletion in Mice Increases Manganese Deposition and Produces Neurotoxic Signatures and Diminished Motor Activity. J. Neurosci..

[35] Aydemir T.B., Thorn T.L., Ruggiero C.H., Pompilus M., Febo M., Cousins R.J. (2020). Intestine-specific deletion of metal transporter Zip14 (Slc39a14) causes brain manganese overload and locomotor defects of manganism. Am. J. Physiol. Gastrointest. Liver Physiol..

[36] Jenkitkasemwong S., Akinyode A., Paulus E., Weiskirchen R., Hojyo S., Fukada T., Giraldo G., Schrier J., Garcia A., Janus C. (2018). SLC39A14 deficiency alters manganese homeostasis and excretion resulting in brain manganese accumulation and motor deficits in mice. Proc. Natl. Acad. Sci. USA.

[37] Thompson K.J., Wessling-Resnick M. (2019). ZIP14 is degraded in response to manganese exposure. Biometals.

[38] Marti-Sanchez L., Ortigoza-Escobar J.D., Darling A., Villaronga M., Baide H., Molero-Luis M., Batllori M., Vanegas M.I., Muchart J., Aquino L. (2018). Hypermanganesemia due to mutations in SLC39A14: Further insights into Mn deposition in the central nervous system. Orphanet. J. Rare Dis..

[39] Zeglam A., Abugrara A., Kabuka M. (2019). Autosomal-recessive iron deficiency anemia, dystonia and hypermanganesemia caused by new variant mutation of the manganese transporter gene SLC39A14. Acta Neurol. Belg..

[40] Chen P., Bowman A.B., Mukhopadhyay S., Aschner M. (2015). SLC30A10: A novel manganese transporter. Worm.

[41] Taylor C.A., Hutchens S., Liu C., Jursa T., Shawlot W., Aschner M., Smith D.R., Mukhopadhyay S. (2015). SLC30A10 transporter in the digestive system regulates brain manganese under basal conditions while brain SLC30A10 protects against neurotoxicity. J. Biol. Chem..

[42] Mercadante C.J., Prajapati M., Conboy H.L., Dash M.E., Herrera C., Pettiglio M.A., Cintron-Rivera L., Salesky M.A., Rao D.B., Bartnikas T.B. (2019). Manganese transporter Slc30a10 controls physiological manganese excretion and toxicity. J. Clin. Investig..

[43] Mukhopadhyay S. (2018). Familial manganese-induced neurotoxicity due to mutations in SLC30A10 or SLC39A14. Neurotoxicology.

[44] Mukhtiar K., Ibrahim S., Tuschl K., Mills P. (2016). Hypermanganesemia with Dystonia, Polycythemia and Cirrhosis (HMDPC) due to mutation in the SLC30A10 gene. Brain Dev..

[45] Steimle B.L., Smith F.M., Kosman D.J. (2019). The solute carriers ZIP8 and ZIP14 regulate manganese accumulation in brain microvascular endothelial cells and control brain manganese levels. J. Biol. Chem..

[46] Lin W., Vann D.R., Doulias P.T., Wang T., Landesberg G., Li X., Ricciotti E., Scalia R., He M., Hand N.J. (2017). Hepatic metal ion transporter ZIP8 regulates manganese homeostasis and manganese-dependent enzyme activity. J. Clin. Investig..

[47] Riley L.G., Cowley M.J., Gayevskiy V., Roscioli T., Thorburn D.R., Prelog K., Bahlo M., Sue C.M., Balasubramaniam S., Christodoulou J. (2017). A SLC39A8 variant causes manganese deficiency, and glycosylation and mitochondrial disorders. J. Inherit. Metab. Dis..

[48] Mealer R.G., Jenkins B.G., Chen C.Y., Daly M.J., Ge T., Lehoux S., Marquardt T., Palmer C.D., Park J.H., Parsons P.J. (2020). The schizophrenia risk locus in SLC39A8 alters brain metal transport and plasma glycosylation. Sci. Rep..

[49] Nebert D.W., Liu Z. (2019). SLC39A8 gene encoding a metal ion transporter: Discovery and bench to bedside. Hum. Gen..

[50] Ling J., Yang S., Huang Y., Wei D., Cheng W. (2018). Identifying key genes, pathways and screening therapeutic agents for manganese-induced Alzheimer disease using bioinformatics analysis. Medicine.

[51] Tian Y., Guo S., Chen C., Zhao L., Li Z., Yan Y. (2018). Gene sequence screening for manganese poisoning-susceptible genes and analysis of gene interaction effects. Environ. Toxicol. Pharmacol..

[52] Pfalzer A.C., Wilcox J.M., Codreanu S.G., Totten M., Bichell T.J.V., Halbesma T., Umashanker P., Yang K.L., Parmalee N.L., Sherrod S.D. (2020). Huntington’s disease genotype suppresses global manganese-responsive processes in pre-manifest and manifest YAC128 mice. Metallomics.

[53] Gandhi D., Sivanesan S., Kannan K. (2018). Manganese-Induced Neurotoxicity and Alterations in Gene Expression in Human Neuroblastoma SH-SY5Y Cells. Biol. Trace Elem. Res..

[54] Tuschl K., White R.J., Valdivia L.E., Niklaus S., Bianco I.H., Sealy I.M., Neuhauss S.C.F., Houart C., Wilson S.W., Busch-Nentwich E.M. (2020). Loss of slc39a14 causes simultaneous manganese deficiency and hypersensitivity in zebrafish. bioRxiv.

[55] Rudgalvyte M., Peltonen J., Lakso M., Nass R., Wong G. (2016). RNA-Seq Reveals Acute Manganese Exposure Increases Endoplasmic Reticulum Related and Lipocalin mRNAs in Caenorhabditis elegans. J. Biochem. Mol. Toxicol..

[56] Mythri R.B., Raghunath N.R., Narwade S.C., Pandareesh M., Sabitha K.R., Aiyaz M., Chand B., Sule M., Ghosh K., Kumar S. (2017). Manganese- and 1-methyl-4-phenylpyridinium-induced neurotoxicity display differences in morphological, electrophysiological and genome-wide alterations: Implications for idiopathic Parkinson’s disease. J. Neurochem..

[57] Hernández R.B., Carrascal M., Abian J., Michalke B., Farina M., Gonzalez Y.R., Iyirhiaro G.O., Moteshareie H., Burnside D., Golshani A. (2020). Manganese-induced neurotoxicity in cerebellar granule neurons due to perturbation of cell network pathways with potential implications for neurodegenerative disorders. Metallomics.

[58] Neth K., Lucio M., Walker A., Zorn J., Schmitt-Kopplin P., Michalke B. (2015). Changes in Brain Metallome/Metabolome Pattern due to a Single i.v. Injection of Manganese in Rats. PLoS ONE.

[59] Wang H., Liu Z., Wang S., Cui D., Zhang X., Liu Y., Zhang Y. (2017). UHPLC-Q-TOF/MS based plasma metabolomics reveals the metabolic perturbations by manganese exposure in rat models. Metallomics.

[60] Fernandes J., Chandler J.D., Liu K.H., Uppal K., Hao L., Hu X., Go Y.M., Jones D.P. (2019). Metabolomic Responses to Manganese Dose in SH-SY5Y Human Neuroblastoma Cells. Toxicol. Sci..

[61] Fernandes J., Chandler J.D., Lili L.N., Uppal K., Hu X., Hao L., Go Y.M., Jones D.P. (2019). Transcriptome Analysis Reveals Distinct Responses to Physiologic versus Toxic Manganese Exposure in Human Neuroblastoma Cells. Front. Genet..

[62] Tarale P., Sivanesan S., Daiwile A.P., Stöger R., Bafana A., Naoghare P.K., Parmar D., Chakrabarti T., Kannan K. (2017). Global DNA methylation profiling of manganese-exposed human neuroblastoma SH-SY5Y cells reveals epigenetic alterations in Parkinson’s disease-associated genes. Arch. Toxicol..

[63] Yang N., Wei Y., Wang T., Guo J., Sun Q., Hu Y., Yan X., Zhu X., Tang B., Xu Q. (2016). Genome-wide analysis of DNA methylation during antagonism of DMOG to MnCl2-induced cytotoxicity in the mouse substantia nigra. Sci. Rep..

[64] Maccani J.Z., Koestler D.C., Houseman E.A., Armstrong D.A., Marsit C.J., Kelsey K.T. (2015). DNA methylation changes in the placenta are associated with fetal manganese exposure. Reprod. Toxicol..

[65] Searles Nielsen S., Checkoway H., Criswell S.R., Farin F.M., Stapleton P.L., Sheppard L., Racette B.A. (2015). Inducible nitric oxide synthase gene methylation and parkinsonism in manganese-exposed welders. Parkinsonism. Relat. Disord..

[66] Guo Z., Zhang Z., Wang Q., Zhang J., Wang L., Zhang Q., Li H., Wu S. (2018). Manganese chloride induces histone acetylation changes in neuronal cells: Its role in manganese-induced damage. Neurotoxicology.

[67] Tarale P., Chakrabarti T., Sivanesan S., Naoghare P., Bafana A., Krishnamurthi K. (2016). Potential Role of Epigenetic Mechanism in Manganese Induced Neurotoxicity. Biomed. Res. Int..

[68] Peres T.V., Arantes L.P., Miah M.R., Bornhorst J., Schwerdtle T., Bowman A.B., Leal R.B., Aschner M. (2018). Role of Caenorhabditis elegans AKT-1/2 and SGK-1 in Manganese Toxicity. Neurotox. Res..

[69] Bryan M.R., Uhouse M.A., Nordham K.D., Joshi P., Rose D.I.R., O’Brien M.T., Aschner M., Bowman A.B. (2018). Phosphatidylinositol 3 kinase (PI3K) modulates manganese homeostasis and manganese-induced cell signaling in a murine striatal cell line. Neurotoxicology.

[70] Bryan M.R., Nordham K.D., Rose D.I.R., O’Brien M.T., Joshi P., Foshage A.M., Gonçalves F.M., Nitin R., Uhouse M.A., Aschner M. (2020). Manganese Acts upon Insulin/IGF Receptors to Phosphorylate AKT and Increase Glucose Uptake in Huntington’s Disease Cells. Mol. Neurobiol..

[71] Tidball A.M., Bryan M.R., Uhouse M.A., Kumar K.K., Aboud A.A., Feist J.E., Ess K.C., Neely M.D., Aschner M., Bowman A.B. (2015). A novel manganese-dependent ATM-p53 signaling pathway is selectively impaired in patient-based neuroprogenitor and murine striatal models of Huntington’s disease. Hum. Mol. Genet..

[72] Parsons-White A.B., Spitzer N. (2018). Environmentally relevant manganese overexposure alters neural cell morphology and differentiation in vitro. Toxicol. In Vitro.

[73] Adamson S.X., Shen X., Jiang W., Lai V., Wang X., Shannahan J.H., Cannon J.R., Chen J., Zheng W. (2018). Subchronic Manganese Exposure Impairs Neurogenesis in the Adult Rat Hippocampus. Toxicol. Sci..

[74] Fu S., Jiang W., Gao X., Zeng A., Cholger D., Cannon J., Chen J., Zheng W. (2016). Aberrant Adult Neurogenesis in the Subventricular Zone-Rostral Migratory Stream-Olfactory Bulb System Following Subchronic Manganese Exposure. Toxicol. Sci..

[75] Fu S., O’Neal S., Hong L., Jiang W., Zheng W. (2015). Elevated adult neurogenesis in brain subventricular zone following in vivo manganese exposure: Roles of copper and DMT1. Toxicol. Sci..

[76] Kikuchihara Y., Abe H., Tanaka T., Kato M., Wang L., Ikarashi Y., Yoshida T., Shibutani M. (2015). Relationship between brain accumulation of manganese and aberration of hippocampal adult neurogenesis after oral exposure to manganese chloride in mice. Toxicology.

[77] Sarkar S., Malovic E., Jin H., Kanthasamy A., Kanthasamy A.G., Aschner M., Costa L. (2019). The role of manganese in neuroinflammation. Advances in Neurotoxicology.

[78] Ke T., Sidoryk-Wegrzynowicz M., Pajarillo E., Rizor A., Soares F., Lee E., Aschner M. (2019). Role of Astrocytes in Manganese Neurotoxicity Revisited. Neurochem. Res..

[79] Popichak K.A., Afzali M.F., Kirkley K.S., Tjalkens R.B. (2018). Glial-neuronal signaling mechanisms underlying the neuroinflammatory effects of manganese. J. Neuroinflamm..

[80] Kirkley K.S., Popichak K.A., Afzali M.F., Legare M.E., Tjalkens R.B. (2017). Microglia amplify inflammatory activation of astrocytes in manganese neurotoxicity. J. Neuroinflamm..

[81] Hammond S.L., Bantle C.M., Popichak K.A., Wright K.A., Thompson D., Forero C., Kirkley K.S., Damale P.U., Chong E., Tjalkens R.B. (2020). NF-κB Signaling in Astrocytes Modulates Brain Inflammation and Neuronal Injury Following Sequential Exposure to Manganese and MPTP During Development and Aging. Toxicol. Sci..

[82] Park E., Chun H.S. (2020). Melatonin Attenuates Manganese and Lipopolysaccharide-Induced Inflammatory Activation of BV2 Microglia. Neurochem. Res..

[83] Yin L., Dai Q., Jiang P., Zhu L., Dai H., Yao Z., Liu H., Ma X., Qu L., Jiang J. (2018). Manganese exposure facilitates microglial JAK2-STAT3 signaling and consequent secretion of TNF-a and IL-1β to promote neuronal death. Neurotoxicology.

[84] Chen J., Su P., Luo W., Chen J. (2018). Role of LRRK2 in manganese-induced neuroinflammation and microglial autophagy. Biochem. Biophys. Res. Commun..

[85] Sarkar S., Malovic E., Harischandra D.S., Ngwa H.A., Ghosh A., Hogan C., Rokad D., Zenitsky G., Jin H., Anantharam V. (2018). Manganese exposure induces neuroinflammation by impairing mitochondrial dynamics in astrocytes. Neurotoxicology.

[86] Zhao X., Yin L., Wu Y., Han M., Zhuang Y., Cong Y., Liu Y., Chen G., Jiang J. (2019). Manganese induces neuroinflammation via NF-κB/ROS NLRP3 pathway in rat brain striatum and HAPI cells. Mol. Cell Toxicol..

[87] Wang D., Zhang J., Jiang W., Cao Z., Zhao F., Cai T., Aschner M., Luo W. (2017). The role of NLRP3-CASP1 in inflammasome-mediated neuroinflammation and autophagy dysfunction in manganese-induced, hippocampal-dependent impairment of learning and memory ability. Autophagy.

[88] Fang Y., Peng D., Liang Y., Lu L., Li J., Zhao L., Ou S., Li S., Aschner M., Jiang Y. (2020). Sodium P-aminosalicylic Acid Inhibits Manganese-Induced Neuroinflammation in BV2 Microglial Cells via NLRP3-CASP1 Inflammasome Pathway. Biol. Trace Elem. Res..

[89] Peng D., Li J., Deng Y., Zhu X., Zhao L., Zhang Y., Li Z., Ou S., Li S., Jiang Y. (2020). Sodium para-aminosalicylic acid inhibits manganese-induced NLRP3 inflammasome-dependent pyroptosis by inhibiting NF-κB pathway activation and oxidative stress. J. Neuroinflamm..

[90] Sarkar S., Rokad D., Malovic E., Luo J., Harischandra D.S., Jin H., Anantharam V., Huang X., Lewis M., Kanthasamy A. (2019). Manganese activates NLRP3 inflammasome signaling and propagates exosomal release of ASC in microglial cells. Sci. Signal..

[91] Wang W., Li D., Ding X., Zhao Q., Chen J., Tian K., Qiu Y., Lu L. (2017). N-Acetylcysteine protects inner ear hair cells and spiral ganglion neurons from manganese exposure by regulating ROS levels. Toxicol. Lett..

[92] Smith M.R., Fernandes J., Go Y.M., Jones D.P. (2017). Redox dynamics of manganese as a mitochondrial life-death switch. Biochem. Biophys. Res. Commun..

[93] Gugnani K.S., Vu N., Rondón-Ortiz A.N., Böhlke M., Maher T.J., Pino-Figueroa A.J. (2018). Neuroprotective activity of macamides on manganese-induced mitochondrial disruption in U-87 MG glioblastoma cells. Toxicol. Appl. Pharmacol..

[94] Bonke E., Siebels I., Zwicker K., Dröse S. (2016). Manganese ions enhance mitochondrial H2O2 emission from Krebs cycle oxidoreductases by inducing permeability transition. Free Radic. Biol. Med..

[95] Neely M.D., Davison C.A., Aschner M., Bowman A.B. (2017). From the Cover: Manganese and Rotenone-Induced Oxidative Stress Signatures Differ in iPSC-Derived Human Dopamine Neurons. Toxicol. Sci..

[96] Warren E.B., Bryan M.R., Morcillo P., Hardeman K.N., Aschner M., Bowman A.B. (2020). Manganese-induced Mitochondrial Dysfunction Is Not Detectable at Exposures Below the Acute Cytotoxic Threshold in Neuronal Cell Types. Toxicol. Sci..

[97] Ueda K., Okamoto Y., Aoki A., Jinno H. (2020). Catecholamine oxidation-mediated transcriptional inhibition in Mn neurotoxicity. J. Toxicol. Sci..

[98] Moyano P., García J.M., García J., Anadon M.J., Naval M.V., Frejo M.T., Sola E., Pelayo A., Pino J.D. (2020). Manganese increases Aβ and Tau protein levels through proteasome 20S and heat shock proteins 90 and 70 alteration, leading to SN56 cholinergic cell death following single and repeated treatment. Ecotoxicol. Environ. Saf..

[99] Deng Y., Zhu J., Mi C., Xu B., Jiao C., Li Y., Xu D., Liu W., Xu Z. (2015). Melatonin antagonizes Mn-induced oxidative injury through the activation of keap1-Nrf2-ARE signaling pathway in the striatum of mice. Neurotox. Res..

[100] Zhang Z., Guo Z., Zhan Y., Li H., Wu S. (2017). Role of histone acetylation in activation of nuclear factor erythroid 2-related factor 2/heme oxygenase 1 pathway by manganese chloride. Toxicol. Appl. Pharmacol..

[101] Jiang J., Shi S., Zhou Q., Ma X., Nie X., Yang L., Han J., Xu G., Wan C. (2014). Downregulation of the Wnt/β-catenin signaling pathway is involved in manganese-induced neurotoxicity in rat striatum and PC12 cells. J. Neurosci. Res..

[102] Culbreth M., Aschner M. (2018). GSK-3β, a double-edged sword in Nrf2 regulation: Implications for neurological dysfunction and disease. F1000Research.

[103] Zhao X., Liu Y., Zhu G., Liang Y., Liu B., Wu Y., Han M., Sun W., Han Y., Chen G. (2019). SIRT1 downregulation mediated Manganese-induced neuronal apoptosis through activation of FOXO3a-Bim/PUMA axis. Sci. Total Environ..

[104] Sun Q., Kang R.R., Chen K.G., Liu K., Ma Z., Liu C., Deng Y., Liu W., Xu B. (2021). Sirtuin 3 is required for the protective effect of Resveratrol on Manganese-induced disruption of mitochondrial biogenesis in primary cultured neurons. J. Neurochem..

[105] Lu J., Zhang H., Chen X., Zou Y., Li J., Wang L., Wu M., Zang J., Yu Y., Zhuang W. (2017). A small molecule activator of SIRT3 promotes deacetylation and activation of manganese superoxide dismutase. Free Radic. Biol. Med..

[106] Bresciani G., da Cruz I.B., González-Gallego J. (2015). Manganese superoxide dismutase and oxidative stress modulation. Adv. Clin. Chem..

[107] Abdel-Magied N., Abdel-Aziz N., Shedid S.M., Ahmed A.G. (2019). Modulating effect of tiron on the capability of mitochondrial oxidative phosphorylation in the brain of rats exposed to radiation or manganese toxicity. Environ. Sci. Pollut. Res..

[108] Fernandes J., Hao L., Bijli K.M., Chandler J.D., Orr M., Hu X., Jones D.P., Go Y.M. (2017). From the Cover: Manganese Stimulates Mitochondrial H_2_O_2_ Production in SH-SY5Y Human Neuroblastoma Cells Over Physiologic as well as Toxicologic Range. Toxicol. Sci..

[109] Li S., Lu L., Liao X., Gao T., Wang F., Zhang L., Xi L., Liu S., Luo X. (2016). Manganese elevates manganese superoxide dismutase protein level through protein kinase C and protein tyrosine kinase. Biometals.

[110] Yoon H., Lee G.H., Li B., Park S.A., Lee S.J., Chae H.J. (2018). Endoplasmic reticulum stress and apoptosis induced by manganese trigger α-synuclein accumulation. Trop. J. Pharm. Res..

[111] Wu C., Yuan G., Mo R., Huang Y., Luo T., Wang J. (2019). Effect of endoplasmic reticulum stress involved in manganese-induced neurotoxicity in rats. Mol. Med. Rep..

[112] Bahar E., Lee G.H., Bhattarai K.R., Lee H.Y., Choi M.K., Rashid H.O., Kim J.Y., Chae H.J., Yoon H. (2017). Polyphenolic Extract of Euphorbia supina Attenuates Manganese-Induced Neurotoxicity by Enhancing Antioxidant Activity through Regulation of ER Stress and ER Stress-Mediated Apoptosis. Int. J. Mol. Sci..

[113] Wang T., Li X., Yang D., Zhang H., Zhao P., Fu J., Yao B., Zhou Z. (2015). ER stress and ER stress-mediated apoptosis are involved in manganese-induced neurotoxicity in the rat striatum in vivo. Neurotoxicology.

[114] Yoon H., Kim D.S., Lee G.H., Kim K.W., Kim H.R., Chae H.J. (2011). Apoptosis Induced by Manganese on Neuronal SK-N-MC Cell Line: Endoplasmic Reticulum (ER) Stress and Mitochondria Dysfunction. Environ. Health Toxicol..

[115] Liu C., Yan D.Y., Wang C., Ma Z., Deng Y., Liu W., Xu B. (2020). IRE1 signaling pathway mediates protective autophagic response against manganese-induced neuronal apoptosis in vivo and in vitro. Sci. Total Environ..

[116] Liu C., Yan D.Y., Wang C., Ma Z., Deng Y., Liu W., Xu B. (2020). Manganese activates autophagy to alleviate endoplasmic reticulum stress-induced apoptosis via PERK pathway. J. Cell Mol. Med..

[117] Liu C., Yan D.Y., Tan X., Ma Z., Wang C., Deng Y., Liu W., Yang T.Y., Xu Z.F., Xu B. (2018). Effect of the cross-talk between autophagy and endoplasmic reticulum stress on Mn-induced alpha-synuclein oligomerization. Environ. Toxicol..

[118] Gorojod R.M., Alaimo A., Porte Alcon S., Pomilio C., Saravia F., Kotler M.L. (2015). The autophagic-lysosomal pathway determines the fate of glial cells under manganese- induced oxidative stress conditions. Free Radic. Biol. Med..

[119] Yan D.Y., Xu B. (2020). The Role of Autophagy in Manganese-Induced Neurotoxicity. Front. Neurosci..

[120] Porte Alcon S., Gorojod R.M., Kotler M.L. (2020). Kinetic and protective role of autophagy in manganese-exposed BV-2 cells. Biochim. Biophys. Acta Mol. Cell Res..

[121] Porte Alcon S., Gorojod R.M., Kotler M.L. (2018). Regulated Necrosis Orchestrates Microglial Cell Death in Manganese-Induced Toxicity. Neuroscience.

[122] Bryan M.R., O’Brien M.T., Nordham K.D., Rose D.I.R., Foshage A.M., Joshi P., Nitin R., Uhouse M.A., Di Pardo A., Zhang Z. (2019). Acute manganese treatment restores defective autophagic cargo loading in Huntington’s disease cell lines. Hum. Mol. Genet..

[123] Ma Z., Wang C., Liu C., Yan D.Y., Deng Y., Liu W., Yang T.Y., Xu Z., Xu B. (2017). The role S-nitrosylation in manganese-induced autophagy dysregulation in SH-SY5Y cells. Environ. Toxicol..

[124] Ma Z., Wang C., Liu C., Yan D.Y., Tan X., Liu K., Jing M.J., Deng Y., Liu W., Xu B. (2020). Manganese induces autophagy dysregulation: The role of S-nitrosylation in regulating autophagy related proteins in vivo and in vitro. Sci. Total Environ..

[125] Zhang Z., Yan J., Bowman A.B., Bryan M.R., Singh R., Aschner M. (2020). Dysregulation of TFEB contributes to manganese-induced autophagic failure and mitochondrial dysfunction in astrocytes. Autophagy.

[126] Yan D., Ma Z., Liu C., Wang C., Deng Y., Liu W., Xu B. (2019). Corynoxine B ameliorates HMGB1-dependent autophagy dysfunction during manganese exposure in SH-SY5Y human neuroblastoma cells. Food Chem. Toxicol..

[127] Vijayan B., Raj V., Nandakumar S., Kishore A., Thekkuveettil A. (2019). Spermine protects alpha-synuclein expressing dopaminergic neurons from manganese-induced degeneration. Cell Biol. Toxicol..

[128] Song D., Ma J., Chen L., Guo C., Zhang Y., Chen T., Zhang S., Zhu Z., Tian L., Niu P. (2017). FOXO3 promoted mitophagy via nuclear retention induced by manganese chloride in SH-SY5Y cells. Metallomics.

[129] Zhang H.T., Mi L., Wang T., Yuan L., Li X.H., Dong L.S., Zhao P., Fu J.L., Yao B.Y., Zhou Z.C. (2016). PINK1/Parkin-mediated mitophagy play a protective role in manganese induced apoptosis in SH-SY5Y cells. Toxicol. In Vitro.

[130] Uribe E., Reyes M.B., Martínez I., Mella K., Salas M., Tarifeño-Saldivia E., López V., García-Robles M., Martínez-Oyanedel J., Figueroa M. (2020). Functional analysis of the Mn^2+^ requirement in the catalysis of ureohydrolases arginase and agmatinase—A historical perspective. J. Inorg. Biochem..

[131] Velázquez-Libera J.L., Caballero J., Tuñón I., Hernández-Rodríguez E.W., Ruiz-Pernía J.J. (2020). On the nature of the enzyme–substrate complex and the reaction mechanism in human Arginase I. A combined molecular dynamics and QM/MM study. ACS Catal..

[132] Madan S., Kron B., Jin Z., Al Shamy G., Campeau P.M., Sun Q., Chen S., Cherian L., Chen Y., Munivez E. (2018). Arginase overexpression in neurons and its effect on traumatic brain injury. Mol. Genet. Metab..

[133] Bichell T.J.V., Wegrzynowicz M., Tipps K.G., Bradley E.M., Uhouse M.A., Bryan M., Horning K., Fisher N., Dudek K., Halbesma T. (2017). Reduced bioavailable manganese causes striatal urea cycle pathology in Huntington’s disease mouse model. Biochim. Biophys. Acta Mol. Basis Dis..

[134] Liu X., Yang J., Lu C., Jiang S., Nie X., Han J., Yin L., Jiang J. (2017). Downregulation of Mfn2 participates in manganese-induced neuronal apoptosis in rat striatum and PC12 cells. Neurochem. Int..

[135] Ding H., Wang F., Su L., Zhao L., Hu B., Zheng W., Yao S., Li Y. (2020). Involvement of MEK5/ERK5 signaling pathway in manganese-induced cell injury in dopaminergic MN9D cells. J. Trace Elem. Med. Biol..

[136] Nkpaa K.W., Onyeso G.I., Kponee K.Z. (2019). Rutin abrogates manganese-Induced striatal and hippocampal toxicity via inhibition of iron depletion, oxidative stress, inflammation and suppressing the NF-κB signaling pathway. J. Trace Elem. Med. Biol..

[137] Yang Y., Ma S., Wei F., Liang G., Yang X., Huang Y., Wang J., Zou Y. (2019). Pivotal role of cAMP-PKA-CREB signaling pathway in manganese-induced neurotoxicity in PC12 cells. Environ. Toxicol..

[138] Zhu G., Liu Y., Zhi Y., Jin Y., Li J., Shi W., Liu Y., Han Y., Yu S., Jiang J. (2019). PKA- and Ca^2+^-dependent p38 MAPK/CREB activation protects against manganese-mediated neuronal apoptosis. Toxicol. Lett..

[139] Yang Y., Wei F., Wang J., Chen R., Zhang J., Li D., Gan D., Yang X., Zou Y. (2020). Manganese modifies Neurotrophin-3 (NT3) and its tropomyosin receptor kinase C (TrkC) in the cortex: Implications for manganese-induced neurotoxicity. Food Chem. Toxicol..

[140] Ma X., Han J., Wu Q., Liu H., Shi S., Wang C., Wang Y., Xiao J., Zhao J., Jiang J. (2015). Involvement of dysregulated Wip1 in manganese-induced p53 signaling and neuronal apoptosis. Toxicol. Lett..

[141] Kim D.S., Jin H., Anantharam V., Gordon R., Kanthasamy A., Kanthasamy A.G. (2017). p73 gene in dopaminergic neurons is highly susceptible to manganese neurotoxicity. Neurotoxicology.

[142] Shi S., Zhao J., Yang L., Nie X., Han J., Ma X., Wan C., Jiang J. (2015). KHSRP participates in manganese-induced neurotoxicity in rat striatum and PC12 cells. J. Mol. Neurosci..

[143] Tong Y., Yang H., Tian X., Wang H., Zhou T., Zhang S., Yu J., Zhang T., Fan D., Guo X. (2014). High manganese, a risk for Alzheimer’s disease: High manganese induces amyloid-β related cognitive impairment. J. Alzheimers Dis..

[144] Wallin C., Kulkarni Y.S., Abelein A., Jarvet J., Liao Q., Strodel B., Olsson L., Luo J., Abrahams J.P., Sholts S.B. (2016). Characterization of Mn(II) ion binding to the amyloid-β peptide in Alzheimer’s disease. J. Trace Elem. Med. Biol..

[145] Lin G., Li X., Cheng X., Zhao N., Zheng W. (2020). Manganese Exposure Aggravates β-Amyloid Pathology by Microglial Activation. Front. Aging Neurosci..

[146] Moyano P., García J.M., Anadon M.J., Lobo M., García J., Frejo M.T., Sola E., Pelayo A., Pino J.D. (2019). Manganese induced ROS and AChE variants alteration leads to SN56 basal forebrain cholinergic neuronal loss after acute and long-term treatment. Food Chem. Toxicol..

[147] Kaur G., Prakash A. (2020). Involvement of the nitric oxide signaling in modulation of naringin against intranasal manganese and intracerbroventricular β-amyloid induced neurotoxicity in rats. J. Nutr. Biochem..

[148] Wu B., Cai H., Tang S., Xu Y., Shi Q., Wei L., Meng L., Zhang N., Wang X., Xiao D. (2020). Methionine-Mediated Protein Phosphatase 2A Catalytic Subunit (PP2Ac) Methylation Ameliorates the Tauopathy Induced by Manganese in Cell and Animal Models. Neurotherapeutics.

[149] Wu B., Cai H., Tang S., Xu Y., Shi Q., Wei L., Meng L., Wang X., Xiao D., Zou Y. (2020). The down-regulation of PP2Ac demethylation attenuates learning and memory impairment in Manganism. Res. Sq..

[150] Mehdizadeh P., Fesharaki S.S.H., Nouri M., Ale-Ebrahim M., Akhtari K., Shahpasand K., Saboury A.A., Falahati M. (2019). Tau folding and cytotoxicity of neuroblastoma cells in the presence of manganese oxide nanoparticles: Biophysical, molecular dynamics, cellular, and molecular studies. Int. J. Biol. Macromol..

[151] Peres T.V., Parmalee N.L., Martinez-Finley E.J., Aschner M. (2016). Untangling the Manganese-α-Synuclein Web. Front. Neurosci..

[152] Wongkongkathep P., Han J.Y., Choi T.S., Yin S., Kim H.I., Loo J.A. (2018). Native Top-Down Mass Spectrometry and Ion Mobility MS for Characterizing the Cobalt and Manganese Metal Binding of α-Synuclein Protein. J. Am. Soc. Mass Spectrom..

[153] Xu B., Liu W., Deng Y., Yang T.Y., Feng S., Xu Z.F. (2015). Inhibition of calpain prevents manganese-induced cell injury and alpha-synuclein oligomerization in organotypic brain slice cultures. PLoS ONE.

[154] Dučić T., Carboni E., Lai B., Chen S., Michalke B., Lázaro D.F., Outeiro T.F., Bähr M., Barski E., Lingor P. (2015). Alpha-Synuclein Regulates Neuronal Levels of Manganese and Calcium. ACS Chem. Neurosci..

[155] Ma Z., Liu K., Li X.R., Wang C., Liu C., Yan D.Y., Deng Y., Liu W., Xu B. (2020). Alpha-synuclein is involved in manganese-induced spatial memory and synaptic plasticity impairments via TrkB/Akt/Fyn-mediated phosphorylation of NMDA receptors. Cell Death Dis..

[156] Sun Y., He Y., Yang L., Liang D., Shi W., Zhu X., Jiang Y., Ou C. (2020). Manganese induced nervous injury by α-synuclein accumulation via ATP-sensitive K(+) channels and GABA receptors. Toxicol. Lett..

[157] Kondru N., Manne S., Hepker M., Malovic E., Jin H., Anantram V., Kanthasamy A., Kanthasamy A.G. (2019). Manganese exposure augments misfolded α-synuclein-induced proinflammatory M1 microglial phenotype and inflammasome activation. FASEB J..

[158] Yan D.Y., Liu C., Tan X., Ma Z., Wang C., Deng Y., Liu W., Xu Z.F., Xu B. (2019). Mn-Induced Neurocytes Injury and Autophagy Dysfunction in Alpha-Synuclein Wild-Type and Knock-Out Mice: Highlighting the Role of Alpha-Synuclein. Neurotox. Res..

[159] Fleming S.M., Santiago N.A., Mullin E.J., Pamphile S., Karkare S., Lemkuhl A., Ekhator O.R., Linn S.C., Holden J.G., Aga D.S. (2018). The effect of manganese exposure in Atp13a2-deficient mice. Neurotoxicology.

[160] Harischandra D.S., Ghaisas S., Rokad D., Zamanian M., Jin H., Anantharam V., Kimber M., Kanthasamy A., Kanthasamy A.G. (2018). Environmental neurotoxicant manganese regulates exosome-mediated extracellular miRNAs in cell culture model of Parkinson’s disease: Relevance to α-synuclein misfolding in metal neurotoxicity. Neurotoxicology.

[161] Harischandra D.S., Rokad D., Neal M.L., Ghaisas S., Manne S., Sarkar S., Panicker N., Zenitsky G., Jin H., Lewis M. (2019). Manganese promotes the aggregation and prion-like cell-to-cell exosomal transmission of α-synuclein. Sci. Signal..

[162] Tarale P., Daiwile A.P., Sivanesan S., Stöger R., Bafana A., Naoghare P.K., Parmar D., Chakrabarti T., Krishnamurthi K. (2018). Manganese exposure: Linking down-regulation of miRNA-7 and miRNA-433 with α-synuclein overexpression and risk of idiopathic Parkinson’s disease. Toxicol. In Vitro.

[163] Harischandra D.S., Jin H., Anantharam V., Kanthasamy A., Kanthasamy A.G. (2015). α-Synuclein protects against manganese neurotoxic insult during the early stages of exposure in a dopaminergic cell model of Parkinson’s disease. Toxicol. Sci..

[164] Bornhorst J., Chakraborty S., Meyer S., Lohren H., Brinkhaus S.G., Knight A.L., Caldwell K.A., Caldwell G.A., Karst U., Schwerdtle T. (2014). The effects of pdr1, djr1.1 and pink1 loss in manganese-induced toxicity and the role of α-synuclein in C. elegans. Metallomics.

[165] Wang C., Xu B., Ma Z., Liu C., Deng Y., Liu W., Xu Z.F. (2017). Inhibition of Calpains Protects Mn-Induced Neurotransmitter release disorders in Synaptosomes from Mice: Involvement of SNARE Complex and Synaptic Vesicle Fusion. Sci. Rep..

[166] Wang C., Ma Z., Yan D.Y., Liu C., Deng Y., Liu W., Xu Z.F., Xu B. (2018). Alpha-Synuclein and Calpains Disrupt SNARE-Mediated Synaptic Vesicle Fusion During Manganese Exposure in SH-SY5Y Cells. Cells.

[167] Wang C., Xu B., Song Q.F., Deng Y., Liu W., Xu Z.F. (2017). Manganese exposure disrupts SNARE protein complex-mediated vesicle fusion in primary cultured neurons. Environ. Toxicol..

[168] Wang T.Y., Ma Z., Wang C., Liu C., Yan D.Y., Deng Y., Liu W., Xu Z.F., Xu B. (2018). Manganese-induced alpha-synuclein overexpression impairs synaptic vesicle fusion by disrupting the Rab3 cycle in primary cultured neurons. Toxicol. Lett..

[169] Lee E., Karki P., Johnson J., Hong P., Aschner M. (2017). Manganese Control of Glutamate Transporters’ Gene Expression. Adv. Neurobiol..

[170] Karki P., Smith K., Johnson J., Aschner M., Lee E.Y. (2015). Genetic dys-regulation of astrocytic glutamate transporter EAAT2 and its implications in neurological disorders and manganese toxicity. Neurochem. Res..

[171] Johnson J., Pajarillo E., Karki P., Kim J., Son D.S., Aschner M., Lee E. (2018). Valproic acid attenuates manganese-induced reduction in expression of GLT-1 and GLAST with concomitant changes in murine dopaminergic neurotoxicity. Neurotoxicology.

[172] Johnson J., Pajarillo E.A.B., Taka E., Reams R., Son D.S., Aschner M., Lee E. (2018). Valproate and sodium butyrate attenuate manganese-decreased locomotor activity and astrocytic glutamate transporters expression in mice. Neurotoxicology.

[173] Karki P., Kim C., Smith K., Son D.S., Aschner M., Lee E. (2015). Transcriptional Regulation of the Astrocytic Excitatory Amino Acid Transporter 1 (EAAT1) via NF-κB and Yin Yang 1 (YY1). J. Biol. Chem..

[174] Karki P., Webb A., Smith K., Johnson J.J., Lee K., Son D.S., Aschner M., Lee E. (2014). Yin Yang 1 is a repressor of glutamate transporter EAAT2, and it mediates manganese-induced decrease of EAAT2 expression in astrocytes. Mol. Cell Biol..

[175] Pajarillo E., Johnson J., Rizor A., Nyarko-Danquah I., Adinew G., Bornhorst J., Stiboller M., Schwerdtle T., Son D.S., Aschner M. (2020). Astrocyte-specific deletion of the transcription factor Yin Yang 1 in murine substantia nigra mitigates manganese-induced dopaminergic neurotoxicity. J. Biol. Chem..

[176] Karki P., Smith K., Johnson J., Aschner M., Lee E. (2015). Role of transcription factor yin yang 1 in manganese-induced reduction of astrocytic glutamate transporters: Putative mechanism for manganese-induced neurotoxicity. Neurochem. Int..

[177] Escalante M., Soto-Verdugo J., Hernández-Kelly L.C., Hernández-Melchor D., López-Bayghen E., Olivares-Bañuelos T.N., Ortega A. (2020). GLAST Activity is Modified by Acute Manganese Exposure in Bergmann Glial Cells. Neurochem. Res..

[178] Qi Z., Yang X., Sang Y., Liu Y., Li J., Xu B., Liu W., He M., Xu Z., Deng Y. (2020). Fluoxetine and Riluzole Mitigates Manganese-Induced Disruption of Glutamate Transporters and Excitotoxicity via Ephrin-A3/GLAST-GLT-1/Glu Signaling Pathway in Striatum of Mice. Neurotox. Res..

[179] Li Z.C., Wang F., Li S.J., Zhao L., Li J.Y., Deng Y., Zhu X.J., Zhang Y.W., Peng D.J., Jiang Y.M. (2020). Sodium Para-aminosalicylic Acid Reverses Changes of Glutamate Turnover in Manganese-Exposed Rats. Biol. Trace Elem. Res..

[180] Rivera-Mancía S., Tristán-López L., Hernández-Díaz K., Rivera-Espinosa L., Ríos C., Montes S. (2020). In vitro inhibition of brain phosphate-activated glutaminase by ammonia and manganese. J. Trace Elem. Med. Biol..

[181] Guan R., Wang T., Chen J., Luo W., Liu M. (2016). The activation of microglia caused by lead and manganese co-exposure induces activation of astrocytes and decrease of glutamine synthetase activity. Xi Bao Yu Fen Zi Mian Yi Xue Za Zhi.

[182] Dimovasili C., Aschner M., Plaitakis A., Zaganas I. (2015). Differential interaction of hGDH1 and hGDH2 with manganese: Implications for metabolism and toxicity. Neurochem. Int..

[183] Wang L., Fu H., Liu B., Liu X., Chen W., Yu X. (2017). The effect of postnatal manganese exposure on the NMDA receptor signaling pathway in rat hippocampus. J. Biochem. Mol. Toxicol..

[184] Ma R.E., Ward E.J., Yeh C.L., Snyder S., Long Z., Gokalp Yavuz F., Zauber S.E., Dydak U. (2018). Thalamic GABA levels and occupational manganese neurotoxicity: Association with exposure levels and brain MRI. Neurotoxicology.

[185] Edmondson D.A., Ma R.E., Yeh C.L., Ward E., Snyder S., Azizi E., Zauber S.E., Wells E.M., Dydak U. (2019). Reversibility of neuroimaging markers influenced by lifetime occupational manganese exposure. Toxicol. Sci..

[186] Casjens S., Dydak U., Dharmadhikari S., Lotz A., Lehnert M., Quetscher C., Stewig C., Glaubitz B., Schmidt-Wilcke T., Edmondson D. (2018). Association of exposure to manganese and iron with striatal and thalamic GABA and other neurometabolites—Neuroimaging results from the WELDOX II study. Neurotoxicology.

[187] Edmondson D.A., Yeh C.L., Hélie S., Dydak U. (2020). Whole-brain R1 predicts manganese exposure and biological effects in welders. Arch. Toxicol..

[188] Ou C.Y., He Y.H., Sun Y., Yang L., Shi W.X., Li S.J. (2019). Effects of Sub-Acute Manganese Exposure on Thyroid Hormone and Glutamine (Gln)/Glutamate (Glu)-γ- Aminobutyric Acid (GABA) Cycle in Serum of Rats. Int. J. Environ. Res. Public Health.

[189] Li S.J., Ou C.Y., He S.N., Huang X.W., Luo H.L., Meng H.Y., Lu G.D., Jiang Y.M., Vieira Peres T., Luo Y.N. (2017). Sodium p-Aminosalicylic Acid Reverses Sub-Chronic Manganese-Induced Impairments of Spatial Learning and Memory Abilities in Rats, but Fails to Restore γ-Aminobutyric Acid Levels. Int. J. Environ. Res. Public Health.

[190] Ou C.Y., Luo Y.N., He S.N., Deng X.F., Luo H.L., Yuan Z.X., Meng H.Y., Mo Y.H., Li S.J., Jiang Y.M. (2017). Sodium P-Aminosalicylic Acid Improved Manganese-Induced Learning and Memory Dysfunction via Restoring the Ultrastructural Alterations and γ-Aminobutyric Acid Metabolism Imbalance in the Basal Ganglia. Biol. Trace Elem. Res..

[191] Yang X., Tan J., Xu X., Yang H., Wu F., Xu B., Liu W., Shi P., Xu Z., Deng Y. (2020). Prepubertal overexposure to manganese induce precocious puberty through GABA_A_ receptor/nitric oxide pathway in immature female rats. Ecotoxicol. Environ. Saf..

[192] Aschner M., Erikson K.M., Herrero Hernández E., Tjalkens R. (2009). Manganese and its role in Parkinson’s disease: From transport to neuropathology. Neuromol. Med..

[193] Robison G., Sullivan B., Cannon J.R., Pushkar Y. (2015). Identification of dopaminergic neurons of the substantia nigra pars compacta as a target of manganese accumulation. Metallomics.

[194] Fan X.M., Luo Y., Cao Y.M., Xiong T.W., Song S., Liu J., Fan Q.Y. (2020). Chronic Manganese Administration with Longer Intervals Between Injections Produced Neurotoxicity and Hepatotoxicity in Rats. Neurochem. Res..

[195] Gubert P., Puntel B., Lehmen T., Fessel J.P., Cheng P., Bornhorst J., Trindade L.S., Avila D.S., Aschner M., Soares F.A.A. (2018). Metabolic effects of manganese in the nematode Caenorhabditis elegans through DAergic pathway and transcription factors activation. Neurotoxicology.

[196] Guilarte T.R., Yeh C.L., McGlothan J.L., Perez J., Finley P., Zhou Y., Wong D.F., Dydak U., Schneider J.S. (2019). PET imaging of dopamine release in the frontal cortex of manganese-exposed non-human primates. J. Neurochem..

[197] Langley M.R., Ghaisas S., Ay M., Luo J., Palanisamy B.N., Jin H., Anantharam V., Kanthasamy A., Kanthasamy A.G. (2018). Manganese exposure exacerbates progressive motor deficits and neurodegeneration in the MitoPark mouse model of Parkinson’s disease: Relevance to gene and environment interactions in metal neurotoxicity. Neurotoxicology.

[198] Peres T.V., Ong L.K., Costa A.P., Eyng H., Venske D.K., Colle D., Gonçalves F.M., Lopes M.W., Farina M., Aschner M. (2016). Tyrosine hydroxylase regulation in adult rat striatum following short-term neonatal exposure to manganese. Metallomics.

[199] Altenhofen S., Wiprich M.T., Nery L.R., Leite C.E., Vianna M.R.M.R., Bonan C.D. (2017). Manganese(II) chloride alters behavioral and neurochemical parameters in larvae and adult zebrafish. Aquat. Toxicol..

[200] Pajarillo E., Rizor A., Son D.S., Aschner M., Lee E. (2020). The transcription factor REST up-regulates tyrosine hydroxylase and antiapoptotic genes and protects dopaminergic neurons against manganese toxicity. J. Biol. Chem..

[201] Kumasaka M.Y., Yajima I., Ohgami N., Ninomiya H., Iida M., Li X., Oshino R., Tanihata H., Yoshinaga M., Kato M. (2017). Manganese-Mediated Decrease in Levels of c-RET and Tyrosine Hydroxylase Expression In Vitro. Neurotox. Res..

[202] Song Q., Deng Y., Yang X., Bai Y., Xu B., Liu W., Zheng W., Wang C., Zhang M., Xu Z. (2016). Manganese-Disrupted Interaction of Dopamine D1 and NMDAR in the Striatum to Injury Learning and Memory Ability of Mice. Mol. Neurobiol..

[203] Criswell S.R., Warden M.N., Searles Nielsen S., Perlmutter J.S., Moerlein S.M., Sheppard L., Lenox-Krug J., Checkoway H., Racette B.A. (2018). Selective D2 receptor PET in manganese-exposed workers. Neurology.

[204] Conley T.E., Beaudin S.A., Lasley S.M., Fornal C.A., Hartman J., Uribe W., Khan T., Strupp B.J., Smith D.R. (2020). Early postnatal manganese exposure causes arousal dysregulation and lasting hypofunctioning of the prefrontal cortex catecholaminergic systems. J. Neurochem..

[205] Beaudin S.A., Strupp B.J., Lasley S.M., Fornal C.A., Mandal S., Smith D.R. (2015). Oral methylphenidate alleviates the fine motor dysfunction caused by chronic postnatal manganese exposure in adult rats. Toxicol. Sci..

[206] Lasley S.M., Fornal C.A., Mandal S., Strupp B.J., Beaudin S.A., Smith D.R. (2020). Early Postnatal Manganese Exposure Reduces Rat Cortical and Striatal Biogenic Amine Activity in Adulthood. Toxicol. Sci..

[207] Adedara I.A., Ego V.C., Subair T.I., Oyediran O., Farombi E.O. (2017). Quercetin Improves Neurobehavioral Performance Through Restoration of Brain Antioxidant Status and Acetylcholinesterase Activity in Manganese-Treated Rats. Neurochem. Res..

[208] Schetinger M.R.C., Peres T.V., Arantes L.P., Carvalho F., Dressler V., Heidrich G., Bowman A.B., Aschner M. (2019). Combined exposure to methylmercury and manganese during L1 larval stage causes motor dysfunction, cholinergic and monoaminergic up-regulation and oxidative stress in L4 Caenorhabditis elegans. Toxicology.

[209] Bowman A.B., Aschner M. (2014). Considerations on manganese (Mn) treatments for in vitro studies. Neurotoxicology.

